# Organotypic Brain Slice Culture Microglia Exhibit Molecular Similarity to Acutely-Isolated Adult Microglia and Provide a Platform to Study Neuroinflammation

**DOI:** 10.3389/fncel.2020.592005

**Published:** 2020-12-21

**Authors:** Alex R. D. Delbridge, Dann Huh, Margot Brickelmaier, Jeremy C. Burns, Chris Roberts, Ravi Challa, Naideline Raymond, Patrick Cullen, Thomas M. Carlile, Katelin A. Ennis, Mei Liu, Chao Sun, Normand E. Allaire, Marianna Foos, Hui-Hsin Tsai, Nathalie Franchimont, Richard M. Ransohoff, Cherie Butts, Michael Mingueneau

**Affiliations:** ^1^Multiple Sclerosis and Neuroimmunology Research Unit, Biogen, Cambridge, MA, United States; ^2^Biogen Postdoctoral Scientist Program, Biogen, Cambridge, MA, United States; ^3^Translational Biology, Biogen, Cambridge, MA, United States; ^4^Genetic and Neurodevelopmental Disorders, Biogen, Cambridge, MA, United States; ^5^MS Immunology Development Unit, Biogen, Cambridge, MA, United States; ^6^Digital & Quantitative Medicine, Biogen, Cambridge, MA, United States

**Keywords:** microglia, neuroinflammation, brain slice culture, LPS, TNF, GM-CSF, cytokine, chemokine

## Abstract

Microglia are central nervous system (CNS) resident immune cells that have been implicated in neuroinflammatory pathogenesis of a variety of neurological conditions. Their manifold context-dependent contributions to neuroinflammation are only beginning to be elucidated, which can be attributed in part to the challenges of studying microglia *in vivo* and the lack of tractable *in vitro* systems to study microglia function. Organotypic brain slice cultures offer a tissue-relevant context that enables the study of CNS resident cells and the analysis of brain slice microglial phenotypes has provided important insights, in particular into neuroprotective functions. Here we use RNA sequencing, direct digital quantification of gene expression with nCounter® technology and targeted analysis of individual microglial signature genes, to characterize brain slice microglia relative to acutely-isolated counterparts and 2-dimensional (2D) primary microglia cultures, a widely used *in vitro* surrogate. Analysis using single cell and population-based methods found brain slice microglia exhibited better preservation of canonical microglia markers and overall gene expression with stronger fidelity to acutely-isolated adult microglia, relative to *in vitro* cells. We characterized the dynamic phenotypic changes of brain slice microglia over time, after plating in culture. Mechanical damage associated with slice preparation prompted an initial period of inflammation, which resolved over time. Based on flow cytometry and gene expression profiling we identified the 2-week timepoint as optimal for investigation of microglia responses to exogenously-applied stimuli as exemplified by treatment-induced neuroinflammatory changes observed in microglia following LPS, TNF and GM-CSF addition to the culture medium. Altogether these findings indicate that brain slice cultures provide an experimental system superior to *in vitro* culture of microglia as a surrogate to investigate microglia functions, and the impact of soluble factors and cellular context on their physiology.

## Introduction

As the CNS-resident immune cell in the parenchyma, microglia have emerged as a promising cellular target in the context of many diseases including Alzheimer's disease (AD) and other neuroinflammatory/neurodegenerative conditions such as multiple sclerosis (MS) (Hemmer et al., 2015; Jansen et al., 2019; Liu et al., 2019; Rostalski et al., 2019; Bellenguez et al., 2020). In contrast to other cell types, such as neurons or peripheral immune cells, well-validated *in vitro* culture methods that would enable insights into microglia function are lacking. This represents a significant barrier to development of meaningful therapeutics that modify microglia function in a disease setting.

Microglia are yolk sac-derived myeloid cells that colonize the nascent CNS prior to the onset of neurogenesis (Ginhoux et al., 2010). In the adult, microglia are critical for maintenance of CNS homeostasis and neuronal health, with the importance of these functions exemplified by the neurodegenerative phenotype that derives from impaired microglia function (Paloneva et al., 2000, 2002). Several gene expression signatures associated with microglia identity, inflammation, proliferation and specific disease models have been determined through meta-analysis of gene expression profiling data (Friedman et al., 2018). Their relevance to human disease remains uncertain (Mathys et al., 2019).

Neuroinflammation frequently involves complex interactions between CNS-resident and peripheral immune cells, including the release of cytokines, chemokines and other soluble factors facilitating cell-cell communication. The complexity of these systems is exemplified by attempts to define the role of TNF in the CNS. In MS patients, TNF levels in the cerebrospinal fluid (CSF) are associated with disease activity in patients (Sharief and Hentges, 1991; Carrieri et al., 1998), and in the mouse model of MS (experimental autoimmune encephalitis, EAE) TNF promotes disease (Ruddle et al., 1990; Baker et al., 1994; Eugster et al., 1998; Gao et al., 2017). However therapeutic attempts to improve MS outcomes through inhibition of TNF signaling proved to instead worsen disease (The Lenercept Multiple Sclerosis Study Group and The University of British Columbia MS/MRI Analysis Group, 1999). So while the role of TNF is well defined in the context of peripheral myeloid cells, its contribution to regulating microglia physiology remains poorly understood. Recent evidence suggests the functions of TNF-induced microglia might not be conserved and, in some instances, contrary to the function attributed to their peripheral counterparts (Gao et al., 2017). One of the reasons underlying this lack of clarity surrounding the role of TNF in the CNS is the lack of tractable models to study the role of TNF *in vitro*.

To overcome the difficulty of manipulating microglia *in vivo* and avoid the confounding contribution of infiltrating peripheral immune cells evident in many *in vivo* neuroinflammatory models we sought to define a reductionist *in vitro* approach to study the effects of disease-relevant soluble mediators on microglia function. Primary microglia cultured on conventional tissue culture plates represent a simple alternative to study response to disease-relevant stimuli, however gene expression profiling identified an epigenetically-driven loss of microglia identity gene expression within hours of transfer to culture plates (Gosselin et al., 2017). In contrast organotypic brain slice cultures derived from primary mouse tissue preserve tissue architecture important for microglia physiological phenotypes and offer the advantage of a tissue-relevant context that have yielded important insights into neuroinflammatory processes (Czapiga and Colton, 1999; Bernardino, 2005; Lossi et al., 2009; Vinet et al., 2012; Hellwig et al., 2015; Masuch et al., 2016a; Saliba et al., 2017; Yousif et al., 2018; Araki et al., 2019; Croft et al., 2019a; Richter et al., 2019; Sheppard et al., 2019). Analogous studies with rat organotypic brain slice cultures have also provided valuable insights regarding the role of microglia during inflammation (Mitrasinovic et al., 2001; Schermer and Humpel, 2002; Ding and Li, 2008; Hall et al., 2009; Benakis et al., 2010; Jung et al., 2013; Nam et al., 2013; Lutz et al., 2015; Ramírez-Sánchez et al., 2015; Papageorgiou et al., 2016; Chong et al., 2018; Magalhães et al., 2018). Mouse brain slice cultures are typically prepared from early post-natal (2–4 days) brain tissue since neural dendritic networks are yet to fully develop at this timepoint, and tissue is therefore more resilient to damage caused by slice preparation. Importantly cultured brain slice microglia exhibit the characteristic ramified resting state morphology observed *in vivo* (Czapiga and Colton, 1999; Davalos et al., 2005; Nimmerjahn et al., 2005; Haynes et al., 2006; Vinet et al., 2012), perhaps supported by contact dependent signaling from surrounding cells, suggesting that this setting may represent a system closer to the *in vivo* state than monocultured primary microglia and cell lines. Despite the evident utility of the model, no cellular or molecular characterization of brain slice microglia has been reported to-date.

In this study, we characterized the changes in microglia phenotype that occur over time in cultured brain slices relative to *in vivo* microglia and leveraged this experimental platform to determine how microglia respond to soluble factors known to be present in the CNS during neuroinflammation.

## Materials and Methods

### Experimental Mice Treatment and Brain Slice Preparation

Studies involving mice were conducted in accordance with the guidelines of the Biogen Institutional Animal Care and Use Committee (IACUC) under protocol #756.

Singly housed timed pregnant female C57BL/6 mice were procured at E15 from Charles River Laboratories (Wilmington, MA). Single housed time pregnant CX3CR1-GFP [B6.129P2(Cg)-*Cx3cr1*^*tm*1*Litt*^/J, cat #005582] mice from Jackson Laboratories were procured at E15 for experiments involving NeuN analysis. Day of birth was noted and brain slices were prepared from P1-4 pups using a Leica VT1200S Vibratome (Leica Biosystems, Wetzlar, Germany) with chilled 4°C solution bath Thermo Accel 500 LT (Cat# 13-875-182; Thermo Fisher Scientific, Waltham, MA). The age range of the neonates used for slice preparation is within the typical age window for the field (Croft et al., 2019b). Moreover, initial experiments compared the microglia immunophenotype by flow cytometry of P1 and P2 slices and no differences were observed. Briefly, pups were decapitated and the brain was harvested and, following removal of the cerebellum, mounted on the cutting disk with a thin layer of Krazy Glue (Cat# KG925; Elmer's Products, Westerville, OH). The cutting disk with mounted brain tissue were secured to the Vibratome platform submerged in chilled cutting solution containing 0.45% glucose (Cat#G68769; Sigma Burlington, MA), 1x Pen/Strep (Cat# 15140-122; Thermo Fisher Scientific, Waltham, MA) in phosphate-buffered saline (Cat# 14190-144; Thermo Fisher Scientific, Waltham, MA). At speed 0.2 mm/s, oscillation 1 mm, 18° angle, slicing was performed anterior to posterior first removing the olfactory bulbs then the first 600 μm of tissue was discarded and then 6 × 300 μm sequential coronal slices were prepared from each brain for subsequent culture (see Supplementary Figure 1).

For *in vivo* lipopolysaccharide (LPS) treatment experiments C57BL/6 female mice (Jackson Laboratories; Bar Harbor, ME) at 10–15 weeks of age were injected *i.p*. with LPS at a dose of 1 mg/kg (*E. coli*, serotype 055:B5, Cat# L4524; MilliporeSigma, Burlington, MA) prepared in phosphate-buffered saline (Cat# 14190-144; Thermo Fisher Scientific, Waltham, MA). After 24 h the mice were sacrificed by CO_2_ asphyxiation. The mice were immediately transcardially perfused with ice-cold phosphate-buffered saline (Cat# 14190-144; Thermo Fisher Scientific, Waltham, MA) containing 3 mM EDTA (Cat# 15575-038; Thermo Fisher Scientific, Waltham, MA) and dissected brain tissue was either snap frozen in liquid nitrogen (stored at −80°C) or harvested for microglia isolation.

### Brain Slice Culture

Brain slices were individually cultured in 1.2 mL brain slice culture medium at 35°C, 5% CO_2_ in 6-well plates (Cat# 3516; Corning Life Sciences, Corning, NY), supported on 0.40 μm Millicell Cell Culture inserts (Cat# PICM03050; MilliporeSigma, Burlington, MA). Brain slice culture medium consists of 50% BME (Cat#21010046; Life Technologies, Carlsbad, CA), 15% exosome-free heat-inactivated horse serum (Cat#2605088; Life Technologies, Carlsbad, CA), 0.25x Hank's BSS (Cat#H4641; MilliporeSigma, Burlington, MA), 2 nM GlutaMAX (Cat#A1286001; Life Technologies, Carlsbad, CA), 0.5% glucose (Cat#G68769; MilliporeSigma, Burlington, MA), 1x Pen/Strep (Cat# 15140-122; Thermo Fisher Scientific, Waltham, MA), 1x N2 supplement (Cat#17502048; Life Technologies, Carlsbad, CA) made up with sterile distilled water. Exosomes were pelleted and removed from the horse serum by ultracentrifugation at 100,000 g for 18 h at 4°C. Brain slice culture medium was replaced thrice per week including one full medium exchange and two half medium exchanges (400 μL removed, replaced with 600 μL due to evaporation); hPDGF (Cat#100-13A; Peprotech, Rocky Hill, NJ) was added at 10 ng/mL final to the medium on each occasion. Timepoints indicated in weeks, with each week constituting 7 ± 1 days in culture with the exception of “1 day” which indicates 22–26 h in culture.

### Immunofluorescent Staining and Analysis

Membranes containing brain slice culture slices were removed from culture medium and washed with phosphate-buffered saline (PBS) (Cat#14190-144; Thermo Fisher Scientific, Waltham, MA). Slices were then fixed with gentle shaking in 4% paraformaldehyde (Cat#15710; Electron Microscopy Sciences, Hatfield, PA) solution in 1x PBS for 1 h at room temperature. Following an overnight wash in PBS at 4°C, fixed slices were blocked at room temperature for 4 h in 2% bovine serum albumin (Cat#05470; Millipore Sigma, Burlington, MA) in 0.5% Triton-x100 (Cat#T8787; Millipore Sigma, Burlington, MA) in 1xPBS (PBST). Fixed slices were washed 3 times for 15 min in PBST and labeled with NeuN (1:500, Cat#24307; Cell Signaling Technologies, Danvers, MA) in 1x PBST for 72 h at 4°C. Slices were washed again 3 times for 15 min in PBST and incubated with AlexaFluor647 donkey anti-rabbit (1:500, Cat#A21206; Thermo-Fisher Scientific, Waltham, MA) for 8 h at room temperature, washed 3 times for 15 min in PBS, and incubated in DAPI solution (1:1,000 dilution of 1 mg/mL DAPI in PBS) (Cat#D1306; Thermo-Fisher Scientific, Waltham, MA) for 1 h. Slices were washed a final time in PBS and labeled with propidium iodide (Cat#P130MP; Thermo-Fisher Scientific, Waltham, MA) according to manufacturer's instructions. Slices were mounted on slides with Prolong Diamond Antifade (Cat#P36961; Invitrogen, Carlsbad, CA).

Slices were imaged with an Intelligent Imaging Innovations Marianas confocal microscope system (3i, Denver, CO) comprised of a Zeiss AxioObserver microscope (Zeiss, Germany), Yokogawa CSU-W1 spinning disc head (Yokogawa Electric, Japan), and Hamamatsu Flash 4.0 sCMOS camera (Hamamatsu Photonics, Japan). NeuN, PI, and DAPI labeled cells were acquired with a 20x air magnification lens. Analysis and identification of NeuN+, PI+ cells, and DAPI+ cells was performed in a 665 × 665 μm region of the cortex of each brain slice. Positive cells were identified using the Spots Wizard with Imaris Software (Oxford Instruments, United Kingdom) with the following thresholds: DAPI-XY Diameter 3.25, NeuN-XY Diameter 7, PI-XY Diameter 3.25. Cells were identified using the spots wizard for each individual channel following by a colocalization wizard with a 5 μm threshold. Slices were analyzed after 3, 7, and 14 days in culture, *n* = 4 slices/condition were analyzed.

### *In vitro* Mixed Glial Cultures

Cortices were dissected from the brains of C57BL/6 neonatal mice at post-natal day 2 and the meninges were removed. The tissue was dissociated in astrocyte culture medium [10% exosome-free heat-inactivated horse serum (Cat#2605088; Life Technologies, Carlsbad, CA), 10% FBS (Cat# 10082-147; Thermo Fisher Scientific, Waltham, MA), 1 mM sodium pyruvate (Cat# 11360-070; Thermo Fisher Scientific, Waltham, MA), 2 mM L-glutamine (Cat# 25030-149; Thermo Fisher Scientific, Waltham, MA), 1x Pen/Strep (Cat# 15140-122; Thermo Fisher Scientific, Waltham, MA) in DMEM (Cat# 11960-040; Thermo Fisher Scientific, Waltham, MA)] by gentle pipetting before the cell suspension was transferred to a T75 culture flask with a total medium volume of 12 mL. The cells were cultured at 37°C, 5% CO_2_ and medium was changed twice over the first week of culture and thereafter as needed based on medium coloration. Microglia were first observed in the culture medium after ~2 weeks. The medium was collected for isolation and analysis of microglia at day 22.

### Brain Slice Treatment

As specified, brain slices were treated with the following agents for 24 h: 100 ng/mL lipopolysaccharide (*E. coli*, serotype 055:B5, Cat# L4524; MilliporeSigma, Burlington, MA), 50 ng/mL TNF (Cat# 34-8321-82; Thermo Fisher Scientific, Waltham, MA), 20 ng/mL GM-CSF (Cat# 576304; BioLegend, San Diego, CA). Doses were chosen to be consistent with previous studies in microglia or macrophages: GM-CSF (Re et al., 2002; Jost et al., 2003; Parajuli et al., 2012; Koshida et al., 2015; Na et al., 2016) and TNF (Bernardino, 2005). At the time of treatment the agents were prepared in brain slice medium and added to a new 6-well plate. The slice insert was transferred into this plate and the slice bathed with the medium followed by the removal of excess from the top of the slice which was returned to the bottom of the well.

### Culture Supernatant Analysis

TNF, IL-6, and IL-10 levels in the slice culture supernatants were determined according to the manufacturer's specifications by V-PLEX MSD assay (Cat# K15048D-1 or K15267D-1; Meso Scale Diagnostics, Rockville, MD) using 50 μL sample volume with 4 h shaking incubation followed by 2 h shaking incubation with the detection antibody solution.

Neurofilament heavy chain levels in the culture supernatant were determined by Neurofilament Heavy (NF-H) Simple Plex assay analyzed on the Ella instrument (ProteinSimple, San Jose, CA) according to the manufacturer's specifications. Supernatant samples were mixed 1:1 with diluent.

### Microglia Isolation

Microglia were isolated from adult brain tissue (mice aged 10–15 weeks) and cultured brain slices by tissue homogenization and Percoll-mediated density separation; all manipulation steps were completed cold on ice, except the layered Percoll spin at 22°C. For whole brain, the tissue was minced using a razor blade (Cat# 71964; Electron Microscopy Sciences, Hatfield, PA). Minced brain tissue or brain slices were then transferred to an ice-cold Wheaton 7 mL dounce homogenizer (Cat# 357542; Wheaton Industries, Millville, NJ); at this step several slices were pooled as required. Using first the “loose” then the “tight” pestle, the tissue was dissociated gently in 5 mL 25 mM HEPES (Cat# 15630-080; Thermo Fisher Scientific, Waltham, MA) in HBSS (Cat# 14175-095; Thermo Fisher Scientific, Waltham, MA) with ~10 strokes for each, washing residual sample from the pestles to maximize yield. The homogenate was centrifuged at 300 g for 5 min. Following aspiration of the supernatant, the pellet was resuspended in 5 mL 70% isotonic Percoll (Cat# 17-0891-01; GE Healthcare, Chicago, IL) prepared with 25 mM HEPES (Cat# 15630-080; Thermo Fisher Scientific, Waltham, MA) in HBSS (Cat# 14175-095; Thermo Fisher Scientific, Waltham, MA). Fifty milliliters of 37% isotonic Percoll (in HEPES/HBSS) was gently layered above and the tubes were centrifuged at 800 g for 25 min, 22°C with an intermediate acceleration speed and the brake set to the lowest active setting. Following centrifugation, microglia localize to the 70/37 interface and were carefully collected in a 2 mL total collection volume using a 1 mL pipette tip and transferred to a new tube, pre-blocked on ice with 1% BSA (Cat# 80-1928; ENZO Life Sciences, Farmingdale, NY) in phosphate-buffered saline (Cat# 14190-144; Thermo Fisher Scientific, Waltham, MA). The cells were washed by adding an additional 8 mL ice-cold HEPES/HBSS and centrifugation at 300 g for 5 min. The supernatant was aspirated and the washed cells were resuspended in ice-cold 100–200 μL HEPES/HBSS or FACS buffer [1% FBS (Cat# 10082-147; Thermo Fisher Scientific, Waltham, MA), 25 mM HEPES (Cat# 15630-080; Thermo Fisher Scientific, Waltham, MA), 0.5 mM EDTA (Cat# 15575-038; Thermo Fisher Scientific, Waltham, MA), in HBSS (Cat# 14175-095; Thermo Fisher Scientific, Waltham, MA)].

### Flow Cytometry and Cell Sorting

All steps were performed on ice, with ice-cold reagents unless otherwise specified. Following isolation, microglia were blocked with 10% TruStain FcX Fc receptor block (Cat# 101320; BioLegend, San Diego, CA) in FACS buffer [1% FBS (Cat# 10082-147; Thermo Fisher Scientific, Waltham, MA), 25 mM HEPES (Cat# 15630-080; Thermo Fisher Scientific, Waltham, MA), 0.5 mM EDTA (Cat# 15575-038; Thermo Fisher Scientific, Waltham, MA), in HBSS (Cat# 14175-095; Thermo Fisher Scientific, Waltham, MA)] for 10 min on ice, then stained for at least 30 min covered on ice with combinations of the following antibodies conjugated to various fluorochromes from BioLegend (San Diego, CA): CD11B (clone: M1/70), CD45 (clone: 30-F11), CX3CR1 (clone: SA011F11), F4/80 (clone: BM8), P2RY12 (clone: S16007D); or from BD Biosciences (Franklin Lakes, NJ): CD44 (clone: IM7); or Abcam (Cambridge, United Kingdom): TMEM119 (clone: 106-6). The cells were washed with FACS buffer and, if required, stained with secondary antibodies for at least 30 min covered on ice, such as streptavidin BV711 (Cat# 405241; BioLegend, San Diego, CA) or anti-rabbit IgG Alexa Fluor 488 (Cat# 406416; BioLegend, San Diego, CA).

For Ki-67 and CD68 detection the cells were first incubated with eBioscience Fixable Viability Dye eFluor 455UV as described (Cat# 65-0868-14; Thermo Fisher Scientific, Waltham, MA), then fixed for 10 min on ice using the eBioscience Foxp3/Transcription Factor Staining Buffer Set (Cat# 00-5523-00; Thermo Fisher Scientific, Waltham, MA) and stained with antibodies for CD68 (clone: FA-11) and Ki-67 (clone: 16A8) from BioLegend (San Diego, CA) prepared in perm buffer for 30 min on ice.

In Supplementary Figure 4, after surface staining cells were fixed with 2% paraformaldehyde (Cat# 15710; Electron Microscopy Sciences, Hatfield, PA) in phosphate-buffered saline (Cat# 14190-144; Thermo Fisher Scientific, Waltham, MA) for 10 min on ice, washed in FACS buffer and then stored covered in foil prior to FACS analysis.

Prior to sample collection, the cells were washed twice in FACS buffer and resuspended in 100–200 μL FACS buffer (100 ng/mL DAPI added to unfixed samples only). Flow cytometric analysis was performed on the BD LSRFortessa (Becton Dickinson, Franklin Lakes, NJ). Flow sorting was performed on the BD FACSAria III (Becton Dickinson, Franklin Lakes, NJ), and microglia were identified as DAPI negative, CD45 intermediate, CD11B high. Cells sorted for downstream RNA isolation were sorted directly into chilled tubes containing cell lysis buffer from the relevant RNA preparation kit.

### RNA Isolation

RNA was prepared according to the manufacturer's instructions using the Qiagen RNeasy Micro RNA kit (Cat# 74004; Qiagen, Hilden, Germany) for brain slices and isolated microglia, or using the Qiagen RNeasy Mini RNA kit (Cat# 74104; Qiagen, Hilden, Germany) for brain tissue. For the homogenization step, brain slice samples were homogenized using the Omni Bead Ruptor 12 (Cat# 19-050A; Omni International, Kennesaw, GA) in 400 μL RLT lysis buffer with 350 μL used as input for RNA isolation; and for whole cerebrum samples homogenization was performed in 5 mL QIAzol lysis reagent (Cat# 79306; Qiagen, Hilden, Germany) with 200 μL used as input for RNA isolation. The purified RNA was eluted in 14 μL RNAase-free water. QC was performed on the Agilent 2100 Bioanalyzer (Agilent Technologies, Santa Clara, CA) using the RNA 6000 Pico Kit (Cat# 5067-1513; Agilent Technologies, Santa Clara, CA). RIN scores for more than 90% samples tested were eight or higher (>75% were nine or higher). Quantitation was performed using the NanoDrop 2000c (Thermo Fisher Scientific, Waltham, MA) or Quant-iT RiboGreen RNA Assay Kit (Cat# R11490; Thermo Fisher Scientific, Waltham, MA), for tissue and microglia samples, respectively.

### Expression Profiling

NanoString nCounter RNA expression profiling was performed according to the manufacturer's instructions with 50–100 ng RNA input using the Mouse Neuroinflammation Panel (Cat# XT-CSO-MNROI1-12; NanoString, Seattle, WA). For profiling of isolated brain slice microglia, input RNA was pre-amplified using the Low RNA Input Kit (Cat# LOW-RNA-48; NanoString, Seattle, WA) and the associated panel-specific primers (Cat# PP-MNROI1-12; NanoString, Seattle, WA). Output RCC file QC and normalization was performed using nSolver Analysis Software (version 4.0; NanoString, Seattle, WA) to generate normalized counts data for further analysis.

Single cell gene expression profiling was performed using the 10x Chromium Controller & Accessory Kit (Cat# PN-120223; 10x Genomics, Pleasanton, CA), Chromium Single Cell 3' Library & Gel Bead Kit v2 (Cat# PN-120237; 10x Genomics, Pleasanton, CA), and Chromium Single Cell A Chip Kit (Cat# PN-120236; 10x Genomics, Pleasanton, CA). Following sorting, microglia samples were prepared to achieve ~1,000 live FACS-sorted cells per sample (each in a single lane of a 10x chip). Libraries were prepared as described by the manufacturer, denatured as recommended and clustered at 14 pM (instead of 18 pM) prior to sequencing to a target depth of ~100k 3' reads per cell on the Illumina HiSeq 2500 using Hi-output flow cell (Ilumina, San Diego, CA). After sequencing, the Cell Ranger pipeline with default parameters was used to process the sequencing data including fastq generation, generating UMI tables, and QC. Each experimental condition was processed separately, and “cell ranger aggregate” function was used to combine the data into one UMI table for further analysis. UMI counts per cell were normalized to 1 million and outlier discrimination was performed using t-SNE.

For population-based microglia RNAseq analysis RNA samples were normalized for target input of 5–10 ng RNA per sample library. SMART-Seq v4 Ultra Low Input RNA Kit (Cat# 634894; Takara Bio USA, Kusatsu, Shiga Prefecture, Japan) was used for cDNA preparation according to the manufacturer's instructions. cDNA was normalized and 150 pg was used as starting material for library construction with the Nextera XT DNA Library Preparation Kit (Cat# FC-131-1002; Ilumina, San Diego, CA) according to the manufacturer's instructions. Libraries were normalized and pooled before clustering HiSeq PE Cluster Kit V4 cBot (Cat# PE-401-4001; Ilumina, San Diego, CA) and sequencing. For **Figure 5** [microglia LPS *in vivo* reference data] parameters were 50 bp paired-end with a Hi-output run on the Illumina HiSeq 2500 (Ilumina, San Diego, CA) at ~18 million reads per sample. For **Figure 6** and Supplementary Figure 7 (cytokine stimulated slice microglia) parameters were 75 bp single end reads on an Illumina NextSeq sequencer (Ilumina, San Diego, CA) at ~9.5 million reads per sample.

For whole brain tissue RNAseq analysis 500 ng RNA input per sample was used for library preparation using the TruSeq RNA Library Prep Kit (Cat# RS-122-2001; Ilumina, San Diego, CA) according to the manufacturer's instructions. Libraries were normalized and pooled before sequencing with 18 pM input for 50 bp single-end reads on rapid mode on the Illumina HiSeq 2500 (Ilumina, San Diego, CA).

### Quantification and Statistical Analysis

Statistical tests, *n* numbers, sample details, group descriptors (mean, standard deviation), and *P*-values and FDR values are annotated in the Figures and detailed in the Figure Legends. Brain slices were allocated to treatment to prevent over-representation of either caudal or rostral slices in any one group. No other randomization methods were employed.

Expression profiling data was analyzed in Qiagen Omicsoft Array Studio (V10.0, Qiagen, Hilden, Germany). The following R packages were implemented through the Array Studio interface: EdgeR (Robinson et al., 2010; McCarthy et al., 2012), and t-SNE (van der Maaten and Hinton, 2008). Differentially expressed genes (DEGs) were identified in Array Studio using the General Linear Model for population-based data and using EdgeR for single cell data. Thresholds of absolute fold-change > 1.5 and *p*-value or false discovery rate (FDR) < 0.05 were consistently applied to identify differentially expressed genes; these cutoffs were also applied to input genelists for pathway analysis (Ingenuity Pathway Analysis software; Qiagen, Hilden, Germany).

In preparation for Louvain clustering analysis, the single-cell RNA-seq data was analyzed using Scanpy (https://scanpy.readthedocs.io/). For quality control, cells with the following characteristics were discarded: expression of mitochondrial genes occupying >5% of total UMI, number of detected genes < 400, and total number of UMI counts > 30,000. Harmony (Korsunsky et al., 2019) was used for data harmonization, with sample IDs as batch, theta = 1, lambda = 1. For clustering of slice culture microglia, the harmonized PCA from Harmony was fed into scanpy.pp.neighbors with n_pcs = 100 and n_neighbors = 20, and then scanpy.tl.louvain was used with resolution = 0.5. For clustering of microglia from all conditions, n_pcs = 50, n_neighbors = 20, and resolution = 0.8 was used. To mitigate potential over-clustering, gene-gene correlation of selected genes was monitored for each pair of clusters, and clusters with high correlation were merged. Genes for this process were selected as follows: For each cluster, the top 100 genes with average UMI > 0.2 and high gene expression specificity score, which is calculated by normalizing average UMI counts of a given gene per cell across all clusters (Skene and Grant, 2016), were selected. The selected gene sets were combined to calculate gene-gene correlation. Clusters with correlation >0.8 were merged, for example, cluster “0” and “3” in a particular run of slice culture microglia presented (the next highest being 0.26). After distinct clusters were identified, cluster identities were inferred from top DEGs using scanpy.tl.rank_genes_groups with method = “wilcoxon,” as well as previously known gene sets from the literature (Keren-Shaul et al., 2017; Friedman et al., 2018).

Gene-level heatmaps display row normalized values using the “RobustCenterScale” algorithm (Array Studio default): the row median is subtracted from each value which is then by scaled by row median absolute deviation. Geneset heatmap analysis displays geneset mean z-score per group: Z-score values across groups for each gene was determined and these values were averaged for the genes in each geneset. Cell type marker genesets were determined from McKenzie et al. (2018) based on gene inclusion cutoffs of grand mean >4 for each cell type: neurons (four genes), microglia (48 genes), astrocytes (eight genes), oligodendrocyte precursor cells (four genes), oligodendrocytes (20 genes), endothelial cells (18 genes). Gene Ontology analysis of LPS DEGs was performed using GOrilla (Eden et al., 2007, 2009) with the up- and down-regulated unique slice microglia and *in vivo* microglia DEGs separately analyzed against the complete NanoString nCounter Mouse Neuroinflammation Panel genelist.

Flow cytometric data analysis was performed in FlowJo V10 (BD Biosciences, Franklin Lakes, NJ).

Analysis and visualization of data not otherwise described was performed using GraphPad Prism 7 (GraphPad Software, San Diego, CA).

### Data Availability

The datasets generated during this study are available through NCBI GEO (GSE154855).

## Results

### The Initial Culture Period Following Brain Slicing Is Associated With Inflammation, Loss of Neuronal Gene Expression and Enhanced Expression of Microglial Genes

**Figure 1:**
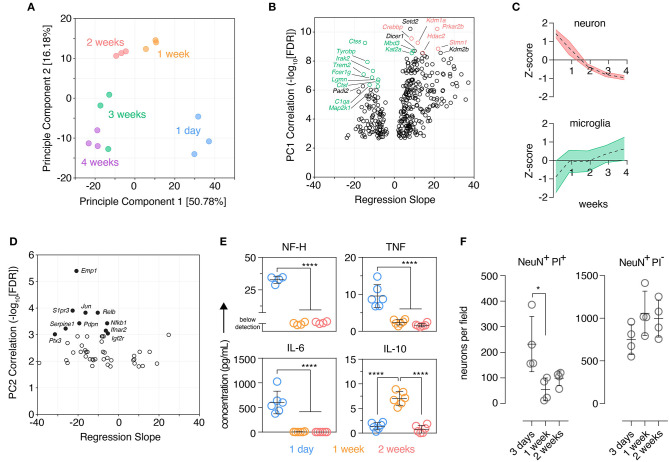
Organotypic brain slices show marks of an early inflammatory reaction that subsides after 2 weeks. Organotypic coronal brain slice cultures were prepared from neonatal mice and cultured *in vitro* on a supporting membrane. **(A)** NanoString expression profiling (Mouse nCounter Neuroinflammation Panel) revealed changes in the tissue transcriptome over time in culture as determined by Principle Component Analysis (PCA). *n* = 3 slices/group. **(B)** Gene-level changes associated with Principle Component 1 were identified by Pearson correlation analysis plotting the statistical significance against scaled effect size (regression slope; model: expression ~ time) to account for different expression levels and prevent down-weighting of low expressed genes. Top 10 positively and negatively correlated genes ranked by *p*-value are labeled; within these genes, those with highest expression in microglia and neurons are indicated in green and red, respectively (Zhang et al., 2014). **(C)** Overall expression changes (z-score normalized) in validated neuronal and microglial marker genes (McKenzie et al., 2018) were plotted over time, mean ± SD. **(D)** Gene-level changes associated with Principle Component 2 were identified by Pearson correlation analysis plotting the statistical significance against scaled effect size (regression slope; model: expression ~ time). Top 10 correlated genes ranked by *p*-value are labeled. **(E)** Protein levels of NF-H, TNF, IL-6, and IL-10 in the culture supernatant was determined at several culture timepoints. *n* = 4–6 slices/group. **(F)** Quantification of dying (PI+) and viable (PI–) neurons (NeuN+) over time in culture identified by immunofluorescence staining. *n* = 4 slice/group, mean ± SD. **** and * denote adjusted *p*-value >0.0001 and 0.05, respectively by one-way ANOVA. See also Supplementary Figure 2.

To characterize the organotypic brain slice system and define its potential to study microglia biology and neuroinflammatory responses, cultured brain slices were harvested following culture periods ranging from 1 day to 4 weeks and subjected to tissue gene expression profiling. By covering a wide range of timepoints our goal was to identify the major periods of change during culture. Principle component (PC) analysis (PCA) revealed sample clustering by timepoint (Figure 1A). Between consecutive timepoints the greatest proportion of the variation along the first principle component (PC1) was observed during the first week of culture with the distance between 1 day and 1 week representing 45% of PC1 range (Figure 1A). Similarly, 97% of the PC2 range was associated with changes occurring during the first week of culture. Differential expression analysis for consecutive timepoints identified 280 differentially expressed genes (DEGs; *p*-value < 0.05, absolute fold-change > 1.5) between 1 day and 1 week, 149 DEGs between 1 and 2 weeks, 100 DEGs between 2 and 3 weeks, and 27 DEGs between 3 and 4 weeks. Altogether, these observations indicated that the largest changes in brain slice gene expression occurred between 1 day and 1 week culture timepoints.

Three hundred sixty-one genes significantly correlated with PC1 were identified by Pearson correlation [false discovery rate (FDR) < 0.001]. Genes negatively-correlated with PC1, i.e., genes that increased over time in culture, were enriched in genes expressed predominantly in microglia with nine of the top 10 most correlated genes exhibiting highest expression in microglia (relative to other CNS cells) based on published cell-type expression data (Zhang et al., 2014) (Figure 1B). Genes positively correlated with PC1, i.e., genes that decreased expression over time in culture were skewed in favor of neuronal genes with five of the top 10 most correlated genes expressed highest in neurons (Zhang et al., 2014) (Figure 1B). Supporting these observations the expression of published cell-type marker genesets (McKenzie et al., 2018) revealed reduced expression of neuronal marker genes over time and, conversely, an increase in expression of microglia marker genes (Figure 1C). Marker genesets for other cell types also changed over time: endothelial cell markers decreased during the first week, oligodendrocyte precursor cell markers transiently increased at 1 week in culture, oligodendrocyte genes increased over time especially beyond 2 weeks, astrocyte marker genes initially increased at 1 week and remained stable thereafter (Supplementary Figure 2). Decreased expression of inflammatory genes characterized the early culture phase of the culture as six out of the top 10 genes that were negatively correlated with PC2 were also associated with inflammatory responses (*Nfkb1, Rela, Jun, Ifnar2, Ptx3, S1pr3*) (Figure 1D). This suggested that brain slicing may be associated with an acute inflammatory phase early in the culture period that is rapidly down-regulated during the first few days of culture.

Consistent with this hypothesis and acute neuronal damage possibly triggering a microglia inflammatory response early in the culture period we detected elevated levels of neurofilament at 1 day, a protein expressed in the nerve fibers of neurons, accompanied by elevated levels of the pro-inflammatory cytokines, IL-6 and TNF (Figure 1E). At 1 week these markers had reached a plateau at a low level and, in contrast, elevated levels of IL-10 were observed, a cytokine typically associated with resolution of inflammation (Burmeister and Marriott, 2018). Immunofluorescence staining showed that a large number of neurons were dying at day 3 post-tissue slicing while neuronal cell death declined at 1 week and remained stable at 2 weeks. Despite this loss of neurons early in the culture period, viable neurons were readily detectable across the culture period (Figure 1F).

**Figure 2:**
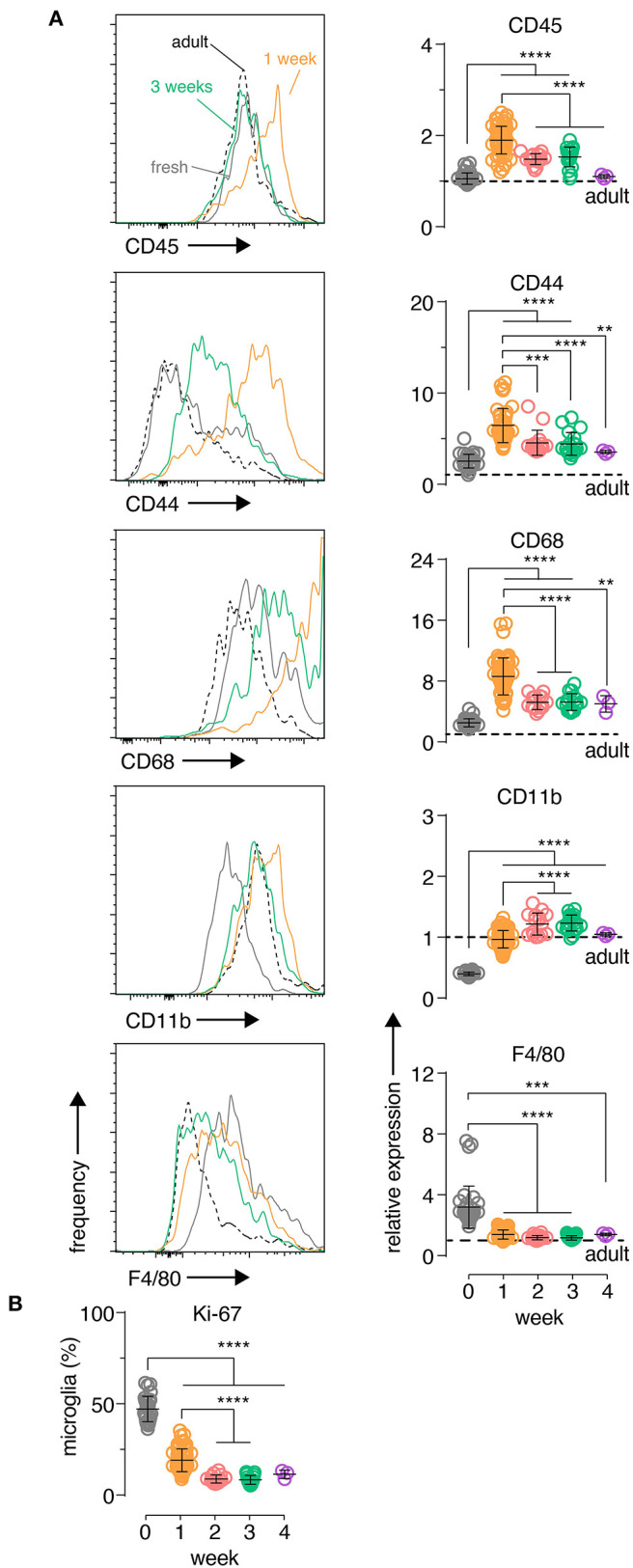
Organotypic brain slice microglia express inflammatory markers at early culture timepoints which then subside after 2 weeks. Organotypic coronal brain slice cultures were prepared from neonatal mice and cultured *in vitro* on a supporting membrane. **(A)** Changes in slice microglia phenotype at 1 week (orange histogram) and 3 weeks (green histogram) compared to fresh uncultured slice microglia (solid gray histogram) and acutely-isolated adult microglia (dashed black histogram) was determined based on the expression of the indicated markers by flow cytometry. Data is quantitated relative to levels in adult *ex vivo* microglia (denoted by the black horizontal dashed line). **(B)** Percentage of Ki-67+ microglia over time for the indicated timepoints. **(A,B)** *n* = 4–59 slices. Pooled data from eight independent experiments. Each symbol represents microglia data from an individual slice, bars indicate mean ± SD. Representative FACS histograms shown, y-axis displays frequency relative to max. ****, *** and ** denote adjusted *p*-value <0.0001, 0.001 and 0.01, respectively by one-way ANOVA. See also Supplementary Figure 3.

To further explore microglia changes at a cellular level, single cell suspensions were prepared from individual brain slices and the expression of several myeloid inflammatory markers were analyzed by flow cytometry in microglia, which were identified by canonical markers CD11B and CD45 (Supplementary Figure 3). CD45, CD44 and CD68 showed an early up-regulation at 1 week in culture, before declining to stable levels closer to those observed in freshly isolated microglia from adult mice (Figure 2A). This period also coincided with a large increase in the percentage of autofluorescent microglia (Supplementary Figure 3), a phenotype associated with an age-dependent accumulation of lysosomal storage bodies containing autofluorescent material and thought to reflect clearance functions of microglia (Burns et al., 2020). CD11B showed a unique behavior with low expression in fresh slices and increasing protein expression early in culture followed by stabilization at adult microglia levels (Figure 2A). Finally, both F4/80 levels (Figure 2A) and the frequency of microglia with a recent history of cell cycling (KI67^+^ microglia; Figure 2B) were elevated initially and then declined over time to levels closer to those observed in adult *ex vivo* microglia. While some of these changes were likely driven by the early inflammatory period following brain slicing such as CD44, CD68, and CD45 (Matsumoto et al., 2012; Bodea et al., 2014; Hendrickx et al., 2017; Rangaraju et al., 2018), this microglia phenotype trajectory was also consistent with the known developmental changes that occur from the neonatal period to adulthood (Nikodemova et al., 2015; Matcovitch-Natan et al., 2016). Importantly, the expression dynamics of all markers indicated that a stable microglia phenotype close to the adult state was established after 2 weeks of culture.

Altogether, these data indicated that inflammatory processes were taking place early during the culture period associated with tissue damage and neuronal process injury, induced by slice preparation for culture. At 2 weeks and beyond, markers of inflammation and developmental progression had subsided or stabilized, respectively, and microglia phenotype was consistent with an adult homeostatic state.

### Brain Slice Microglia Are Transcriptionally Closer to Acutely-Isolated Microglia Than Microglia Cultured *in vitro*

**Figure 3:**
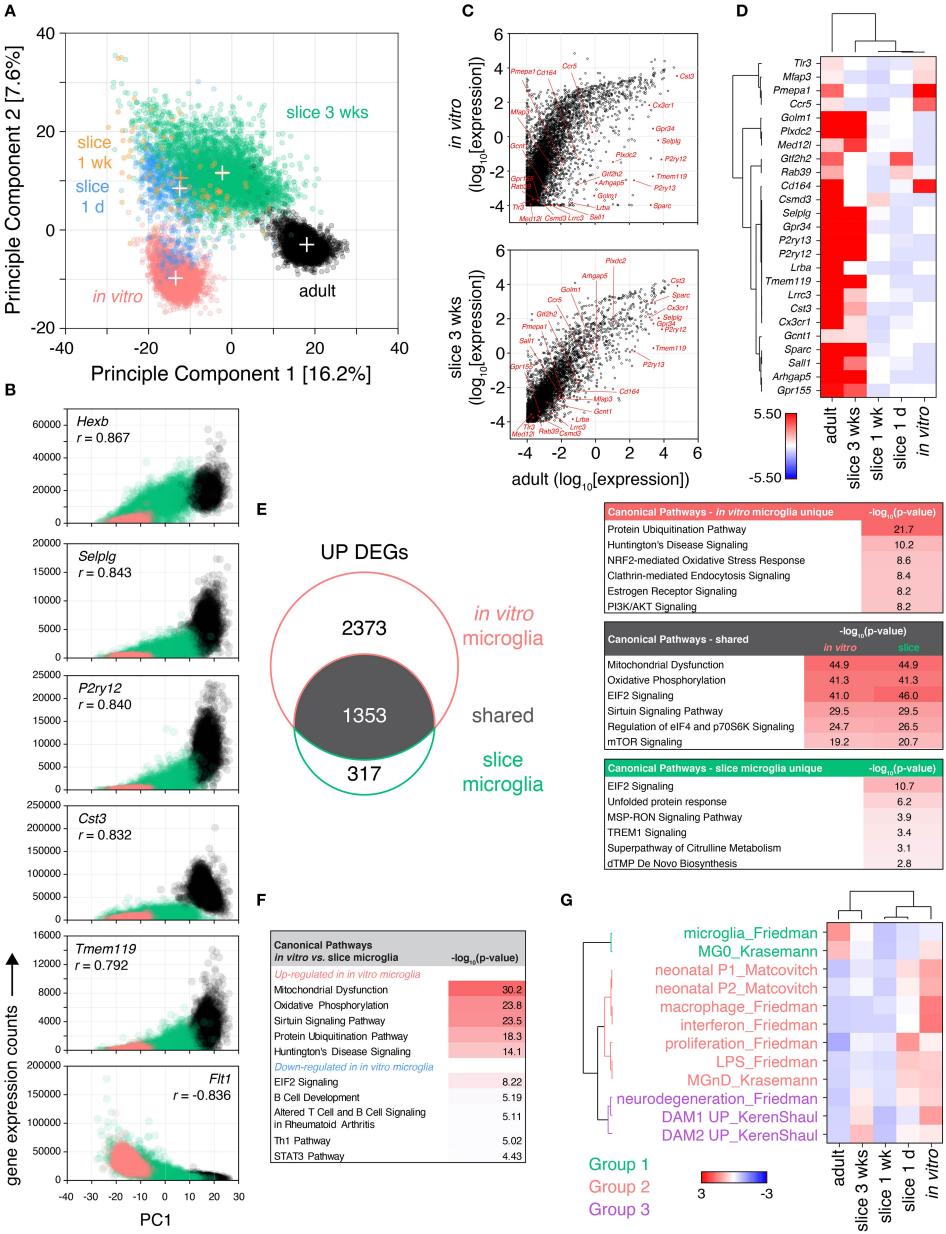
Microglia isolated from cultured brain slices share greater similarity to their adult *in vivo* counterparts than *in vitro* primary microglia. Brain slice culture, *in vitro* primary/mixed glial culture and freshly-isolated adult microglia were isolated by FACS and subjected to single cell RNA expression profiling. **(A)** PCA was performed to determine similarity between cells. “+” indicate cluster centroids (PC1, PC2): *in vitro* (−13.5, −9.8), slice 1 day (−12.6, 8.5), slice 1 week [−12.4, 10.5; (orange) for clarity], slice 3 weeks (−2.3, 11.5), adult (17.1, −3.0). Each circle represents an individual cell, each cluster consists of cells from two independent samples. **(B)** Gene correlation vs. PC1 was performed comparing adult, *in vitro* and slice microglia at 3 weeks to provide insight into genes associated with adult *in vivo* microglia phenotype; normalized gene expression relative to PC1 for the top 6 genes ranked by Pearson correlation coefficient (*r*) shown. **(C)** Mean absolute gene expression was determined for each cluster and compared between slice microglia at 3 weeks, *in vitro* microglia and adult microglia; microglia identity genes are labeled (Friedman et al., 2018). Each symbol represents an individual gene. **(D)** Heatmap representation of mean microglia identity gene expression (Friedman et al., 2018) between microglia clusters. Scale represents median absolute deviation by row (see Methods). **(E)** Up-regulated differentially expressed genes (UP DEGs) between either slice microglia at 3 weeks or *in vitro* microglia relative to adult microglia were compared. DEGs were subjected to Ingenuity Pathway Analysis to identify enrichment of the DEGs in canonical pathways. Top pathways ranked by *p*-value shown for DEGs shared or unique to slice 3 weeks and *in vitro* microglia, relative to adult microglia. **(F)** Pathway analysis was performed separately for genes up-regulated and down-regulated in *in vitro* microglia relative to slice microglia at 3 weeks, top five canonical pathways ranked by *p*-value for each shown. **(G)** Microglia transcriptional differences in culture over time were compared to published genesets associated with specific microglia states and phenotypes (Matcovitch-Natan et al., 2016; Keren-Shaul et al., 2017; Krasemann et al., 2017; Friedman et al., 2018). Geneset mean of z-score normalized gene expression. Sample microglia counts for 1 week slice microglia samples were 33 and 60 cells, while for other samples cell counts were 331–3,517 cells. Further analysis details contained in methods. See also Supplementary Figures 4, 5, and Supplementary Tables 4–6.

To more thoroughly characterize the phenotype of microglia at different timepoints in brain slice culture, we performed single cell gene expression profiling on FACS-purified microglia from brain slices. Timepoints were selected to provide insight into the dynamic changes in phenotype observed during the first 2 weeks of culture relative to the more stable phenotype that was observed after 2 weeks. Slice microglia isolated after 1 day, 1 week, and 3 weeks in culture were compared to microglia isolated from adult mouse brain, and to primary *in vitro*-derived microglia from *in vitro* mixed glial cultures which are widely used *in vitro* as a surrogate for *in vivo* microglia (Figure 3A).

By principle component analysis (PCA), individual microglia separated into distinct clusters according to their sample identity (Figure 3A). The component explaining the majority of the variance in the dataset (PC1) most prominently distinguished *in vitro* from adult microglia, with the slice microglia occupying an intermediate position along PC1. The second principle component uniquely distinguished slice microglia from both adult and *in vitro*-derived microglia. Proximity to the adult microglia cluster was also associated with culture duration with the early culture slice microglia at 1 day and 1 week positioned closer to the *in vitro* cluster while slice microglia at 3 weeks positioned closer to adult microglia.

Further insight into the transcriptional differences driving this separation along PC1 was obtained by identifying the genes most correlated with PC1 based on Pearson correlation analysis (Figure 3B). One hundred twenty genes were identified with *p*-value < 0.001 and absolute correlation coefficient of > 0.5. When the significantly correlated genes were ranked by absolute correlation, five of the top six most correlated genes were genes associated with microglia identity (Butovsky et al., 2014; Keren-Shaul et al., 2017; Krasemann et al., 2017; Friedman et al., 2018): *Tmem119, P2ry12, Hexb, Selplg*, and *Cst3* (Figure 3B). Additional microglia identity genes (*Cx3cr1, Csf1r, Gpr34, Ctss*) were in the top 20 ranked genes. These genes exhibited highest expression in adult microglia, moderate expression in slice microglia, and low/absent expression in *in vitro* microglia. The 4th most correlated gene, *Flt1*, was negatively correlated with PC1 (with high expression in *in vitro* cells, lower in the slice microglia and absent in adult microglia). *Flt1*, encoding VEGFR1, is highly expressed in peripheral monocytes and some tissue-resident macrophages such as lung and liver macrophages, with minimal, if any, expression in adult microglia (Heng et al., 2008). Fifty-five genes were found to be correlated with the second principle component (PC2) with *p*-value < 0.001 and absolute correlation coefficients > 0.5. Of these 69% (38/55) encoded members of the RPL and RPS ribosomal protein family. These genes were up-regulated possibly indicating elevated rates of protein translation in brain slice microglia.

When analyzing absolute gene expression levels, there was a tighter correlation between the adult and 3 week slice microglia transcriptomes than between adult and *in vitro* microglia, including the expression of microglia identity genes (Friedman et al., 2018) (Figure 3C). The relative expression of the latter genes was highest in adult microglia while the majority of them were also highly expressed in 3 week slice microglia, in contrast to the low expression levels observed in *in vitro* microglia and in the slice microglia at early timepoints (1 day and 1 week) (Figure 3D). Despite high relative gene expression, the protein level of CX3CR1 observed in slice microglia was only 56–65% of the adult microglia expression level, while the levels of P2RY12 and TMEM119 were substantially reduced (Supplementary Figure 4). This observation is consistent with the known developmental regulation of *Cx3cr1, P2ry12*, and *Tmem119* gene expression with reported mRNA levels in neonatal P3 microglia of 81, 38, and 21% of adult levels, respectively (Matcovitch-Natan et al., 2016).

Differential expression analysis (FDR < 0.05, absolute fold-change > 1.5) comparing *in vitro* microglia relative to adult microglia identified 4,213 DEGs (3,726 up- and 487 down-regulated), while comparing slice microglia at 3 weeks to adult microglia identified 2011 DEGs (1,670 up- and 341 down-regulated) (Figure 3E, Supplementary Figure 5). Among the total number of 4,043 up-regulated and 598 down-regulated genes pooled across both comparisons, 1,353 and 230 were shared DEGs, respectively (Figure 3E, Supplementary Figure 5). Amongst the shared up-regulated DEGs, pathway analysis identified associations with “Mitochondrial Dysfunction” (*p* < 10^−44^), “Oxidative Phosphorylation” (*p* < 10^−41^), “EIF2 Signaling” (*p* < 10^−41^), “Sirtuin Signaling Pathway” (*p* < 10^−29^), “Regulation of eIF4 and p70S6K Signaling” (*p* < 10^−24^); identified based on enrichment of differentially expressed ATP synthase and electron transport chain complex genes within these pathways (Figure 3E). For the DEGs uniquely up-regulated in the slice microglia there was relatively little enrichment in specific pathways with the exception of “EIF2 Signaling” (*p* < 10^−10^) based on higher expression of ribosomal protein genes. This observation was consistent with the PCA findings and the correlation of ribosomal protein genes with PC2 (Figure 3A). For the DEGs uniquely associated with *in vitro* microglia the top canonical pathway results were “Protein Ubiquitination Pathway” (*p* < 10^−21^), “Huntington's Disease Signaling” (*p* < 10^−10^), and “NRF2-mediated Oxidative Stress Response” (*p* < 10^−8^). These associations were due to gene families encoding heat shock proteins, proteasome/ubiquitin genes, MAPK genes, and RNA polymerase II components, which were collectively indicative of higher levels of protein stress in *in vitro* microglia relative to the slice and adult microglia. In contrast, the down-regulated genes showed limited enrichment of canonical pathways (*p* > 10^−6^; Supplementary Figure 5).

Direct head-to-head comparison of gene expression between *in vitro* microglia and 3-week slice microglia identified 4,216 up-regulated and 1,404 down-regulated DEGs. Pathway analysis of these genes identified up-regulation in *in vitro* microglia of genes associated with “Mitochondrial Dysfunction” (*p* < 10^−30^), “Oxidative Phosphorylation” (*p* < 10^−23^), “Sirtuin Signaling Pathway” (*p* < 10^−23^), “Protein Ubiquitination Pathway” (*p* < 10^−18^) (top four pathways by *p*-value; Figure 3F). Genes down-regulated in *in vitro* microglia were less significant and associated with pathways such as “EIF2 Signaling” (*p* < 10^−8^), “B Cell Development” (*p* < 10^−5^), “Altered T Cell and B Cell Signaling in Rheumatoid Arthritis” (*p* < 10^−5^), “Th1 Pathway” (*p* < 10^−5^) (top four pathways by *p*-value; Figure 3F). The association with these immune-related pathways was driven by lower expression of MHC genes and immune signaling receptors.

To characterize phenotypic changes in slice microglia over time, we determined the change in expression of published microglia gene signatures (Matcovitch-Natan et al., 2016; Keren-Shaul et al., 2017; Krasemann et al., 2017; Friedman et al., 2018) (Figure 3G). Based on normalized expression changes within each geneset, the signatures clustered in three groups (Figure 3G). Adult microglia strongly expressed microglia identity signatures (Group 1) and no other signatures. *In vitro* microglia as well as slice microglia at 1 day exhibited the opposite pattern with no or low expression of microglia identity genes, but high expression of inflammatory, proliferative and neonatal signatures (Group 2), as well as, neurodegenerative signatures (Group 3) (Figure 3G). This observation was consistent with tissue transcriptomics and flow cytometry data (Figures 1, 2). Over time in culture inflammatory and neonatal gene expression signatures in Group 2 decreased with the levels seen at 1 and 3 weeks close to those observed in adult microglia. At 3 weeks increased expression of microglia identity signatures (Group 1) was seen, relative to 1 day and 1 week, despite remaining low compared to adult levels. Also at 3 weeks, we observed increased expression of gene signatures from Group 3 associated with neurodegeneration, including the disease-associated microglia (DAM) phenotype.

**Figure 4:**
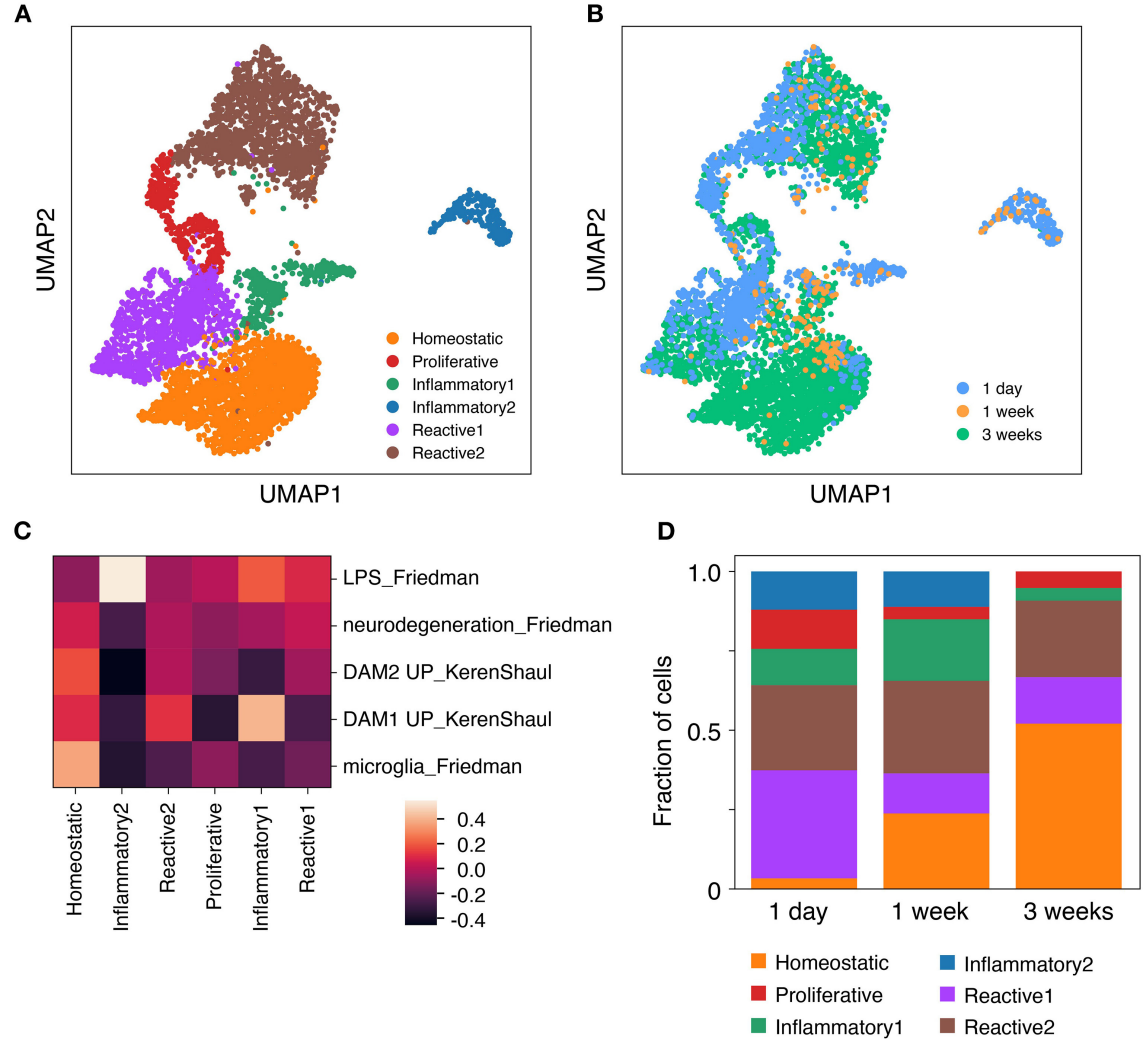
Microglia isolated from cultured brain slices consist of several distinct functional clusters. Brain slice culture, *in vitro* primary/mixed glial culture and freshly-isolated adult microglia were isolated by FACS and subjected to single cell RNA expression profiling. **(A)** UMAP plot of Louvain clusters. **(B)** UMAP plot annotated with microglia sample identity. **(C)** Expression profile for each cluster was compared to published genesets associated with specific microglia states and phenotypes (Keren-Shaul et al., 2017; Friedman et al., 2018). Geneset mean of *z*-score normalized gene expression. **(D)** Cluster composition of brain slice microglia by timepoint. Sample microglia counts for 1 week slice microglia samples were 33 and 60 cells, while for other samples cell counts were 331–3,517 cells. Further analysis details contained in methods. See also Supplementary Figure 6 and Supplementary Table 1.

To examine potential cellular heterogeneity within the slice microglia across timepoints we performed Louvain clustering (Figure 4A). This approach identified six distinct clusters each including cells from multiple timepoints (Figure 4B). To infer the differentiating properties of each cluster, genes that showed enriched expression in each cluster relative to the pooled cells from the other clusters were identified (Supplementary Table 1). This analysis revealed a “homeostatic” microglia cluster that showed enriched expression of canonical homeostatic microglia genes amongst the most significantly enriched genes, among which were in order of decreasing significance: *Selplg, P2ry12, Hexb, Sparc, Olfml3, Cst3, Cd81, C1qc, Cfh, Cx3cr1, Tmem119, C1qa, Gpr34*. To further determine the phenotype of the remaining clusters enriched genes for each were subjected to pathway analysis. This analysis identified cluster 3 as a proliferative cluster enriched in genes associated with cell cycle pathways. Expression of *Mki67* (encoding Ki67) was also uniquely enriched in this cluster, in agreement with flow cytometry data (Figure 2). The remaining clusters were associated with inflammatory genes and pathways, but were more challenging to characterize with confidence. Aided by expression analysis of microglia gene signatures (Keren-Shaul et al., 2017; Friedman et al., 2018), two of these clusters were annotated as “inflammatory” based on high expression of immune signaling pathways and LPS-related inflammatory pathways, while the other two clusters had higher levels of neurodegeneration associated gene expression and were termed “reactive” (Figure 4C).

The proportional representation of these clusters differed across brain slice culture timepoints. While a higher proportion of the homeostatic microglia cluster were observed at 1 week and 3 week timepoints, the reactive and inflammatory clusters reached their peak at 1 day and 1 week timepoints, respectively (Figure 4D).

A similar Louvain clustering-based analysis including also *in vitro* microglia facilitated a more nuanced understanding of the slice microglia phenotype relative to the *in vitro* microglia cells (Supplementary Figure 6). This analysis indeed confirmed very limited cluster overlap between *in vitro* and slice microglia and that the limited number of slice microglia which overlapped with the *in vitro* microglia belonged to either inflammatory, reactive or proliferative clusters (Supplementary Figure 6).

Altogether, analysis of transcriptome-wide expression and of select microglia identity genes and signatures revealed a higher degree of concordance of gene expression between adult microglia and slice microglia at 3 weeks relative to the more limited concordance observed between adult and *in vitro* microglia. Furthermore, in slice microglia at early timepoints we observed high expression of neonatal and inflammatory gene signatures that subsided after 1 week while neurodegeneration-associated genes increased late in the culture period at 3 weeks. Based on these findings and the changes in microglia markers over time (Figures 1, 2) we propose that the 2 week culture timepoint represents the optimal treatment timepoint to explore microglia responses and functions.

### The *in vivo* Microglia LPS Response Is Largely Preserved in Slice Cultures

We next examined whether the ability of microglia to mount an inflammatory response was also preserved in the slice culture environment. After 2 weeks in culture brain slices were treated with the pro-inflammatory agent, lipopolysaccharide (LPS).

**Figure 5:**
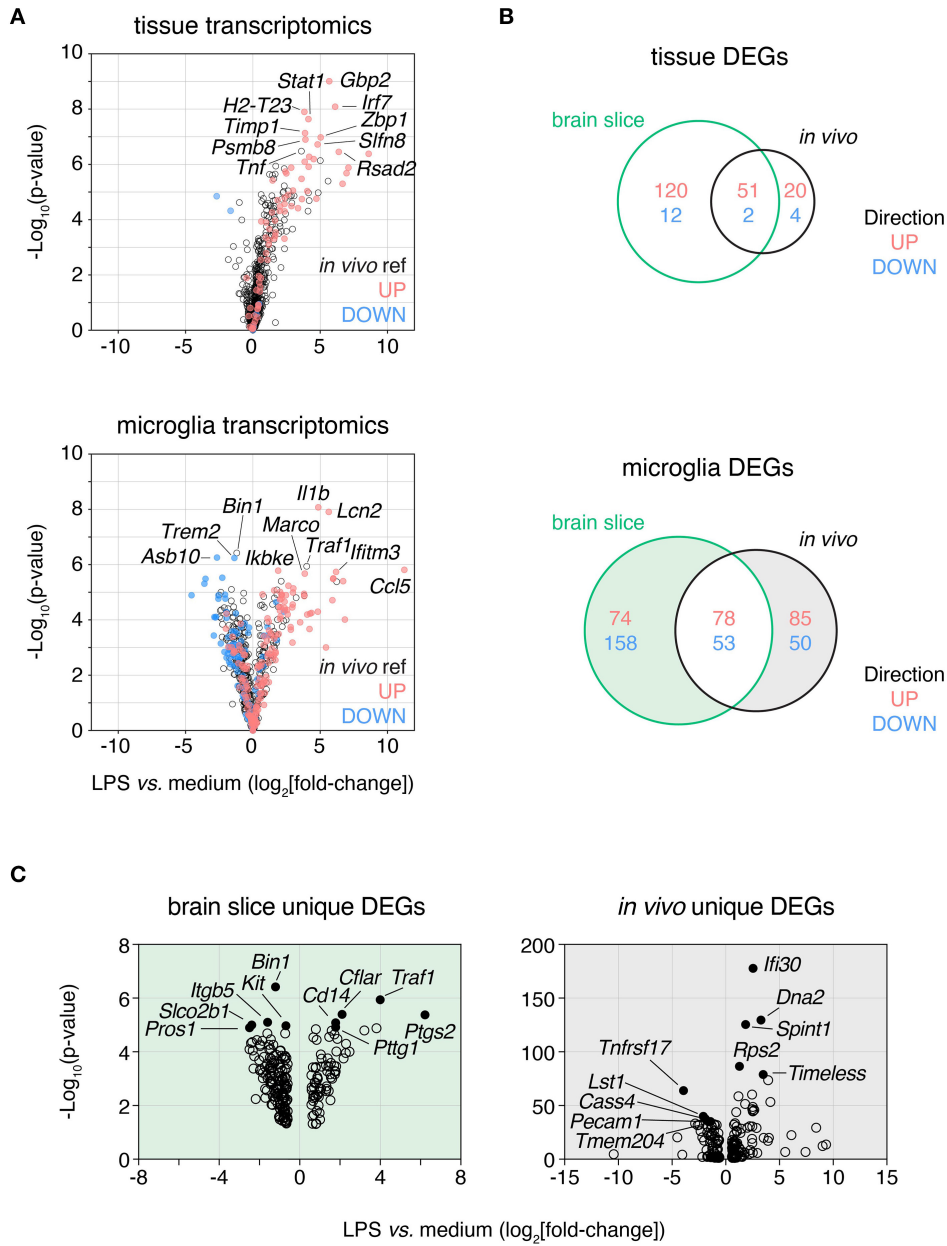
LPS response in brain slices is consistent with *in vivo* response both at a tissue level and in isolated microglia. Slices were treated with lipopolysaccharide (LPS, 100 ng/mL) for 24 h and either whole tissue or FACS-isolated microglia were harvested and subjected to expression profiling across a panel of neuroinflammatory genes (NanoString nCounter Neuroinflammation panel). These data were compared to analogous *in vivo* LPS response RNAseq data derived from either brain tissue or isolated microglia. **(A)** Whole slice (top) and FACS-isolated slice microglia (bottom) LPS response represented as volcano plots (all genes) with up- and down-regulated genes in *in vivo* LPS reference datasets displayed in red and blue, respectively. Top 10 DEGs ranked by *p*-value labeled. **(B)** Venn diagram of LPS-induced DEGs identified at the tissue-level (top) or microglia-level (bottom) after LPS treatment in brain slices or *in vivo*. **(C)** Volcano plots of DEGs unique to either brain slice microglia or *in vivo* microglia (as indicated by shading in **B**). Top five most significantly DE up- and down-regulated genes for each are labeled and indicated in closed circles. *n* = 4 whole slice samples per group; *n* = 3 samples per group for isolated slice microglia (three slices were pooled prior to sorting for each sample). *n* = 4 samples/group from independent mice for LPS *in vivo* reference data. LPS DEGs identified based on absolute fold-change > 1.5 and adj. *p*-value < 0.05. Reference *in vivo* DEGs not included in the NanoString nCounter Neuroinflammation panel are not shown. See also Supplementary Table 2.

Robust gene expression changes were observed in brain slices following treatment of cultures for 24 h with LPS both at slice tissue level and microglia cellular level (Figure 5A). These changes were largely consistent with whole brain tissue and cellular microglia gene expression changes induced *in vivo* following intraperitoneal LPS treatment. Sixty-nine and forty-nine percent of the *in vivo* LPS-induced DEGs were conserved in LPS response in whole brain slice tissue and microglia isolated from brain slices, respectively (Figures 5A,B). It is noteworthy that this similarity in response was observed despite different stimulation paradigms in the two contexts: peripheral injection of LPS for the *in vivo* dataset associated with both CNS vs. peripheral responses inducing direct vs. indirect effects, in contrast to direct application of LPS to the brain slice.

We next examined the DEGs uniquely induced by LPS in slice microglia (232 genes) or in the *in vivo* context (135 genes) to understand the differences in response between the two experimental settings (Supplementary Table 2). By *p*-value the top 10 up-regulated DEGs that changed only in the slice (but not *in vivo*) included genes associated with cell death and inflammatory receptor signaling (*Traf1, Cflar, Bnip3, Tnfrsf1b, Cd14*) and immune cell recruitment (*Ptgs2, Cxcl9, Hdc*). The top 10 up-regulated DEGs (by adj. *p*-val) that changed only *in vivo* (but not in brain slice) included genes associated with cell division (*Dna2, Timeless, Kif2c*), protein production (*Rps2, Calr*) and inflammatory processes (*Ifi30, Spint1, Cst7, Ifitm2, Arhgap24*) (Figure 5C). The top down-regulated genes were less thematically consistent. Due to the small number of DEGs and limitations of the curated geneset in the NanoString panel we were unable to perform robust pathway analysis. With this caveat in mind based on GO term enrichment analysis we observed enrichment within the up-regulated DEGs of the slice microglia suggestive of increased inflammatory activity (top GO term included response to bacterium) and reduced activity of cell cycle pathways for down-regulated DEGs (top GO term included spindle organization). As for the *in vivo* microglia unique up-regulated DEGs, we observed enrichment suggestive of increased cell cycle pathway activity (top 5 processes associated with DNA replication and macromolecule biosynthesis) while unique down-regulated DEGs showed limited enrichment (two GO terms associated with glycosylation).

Altogether, these results indicated that while most of the *in vivo* microglia transcriptional response to LPS was conserved in slice microglia, there were also differences suggesting that LPS was potentially more pro-inflammatory and less pro-mitotic in the brain slice culture system than in the *in vivo* context. More broadly we concluded that the slice culture platform represented a promising means for investigating microglia responses to stimuli associated with neuroinflammatory disease conditions and thereby could facilitate better understanding of microglia biology not easily interrogated *in vivo*.

### TNF and, to a Limited Extent, GM-CSF Induce Expression of Migration/Chemotaxis and Immune Activation Genes While Suppressing Proliferation-Associated Genes

**Figure 6:**
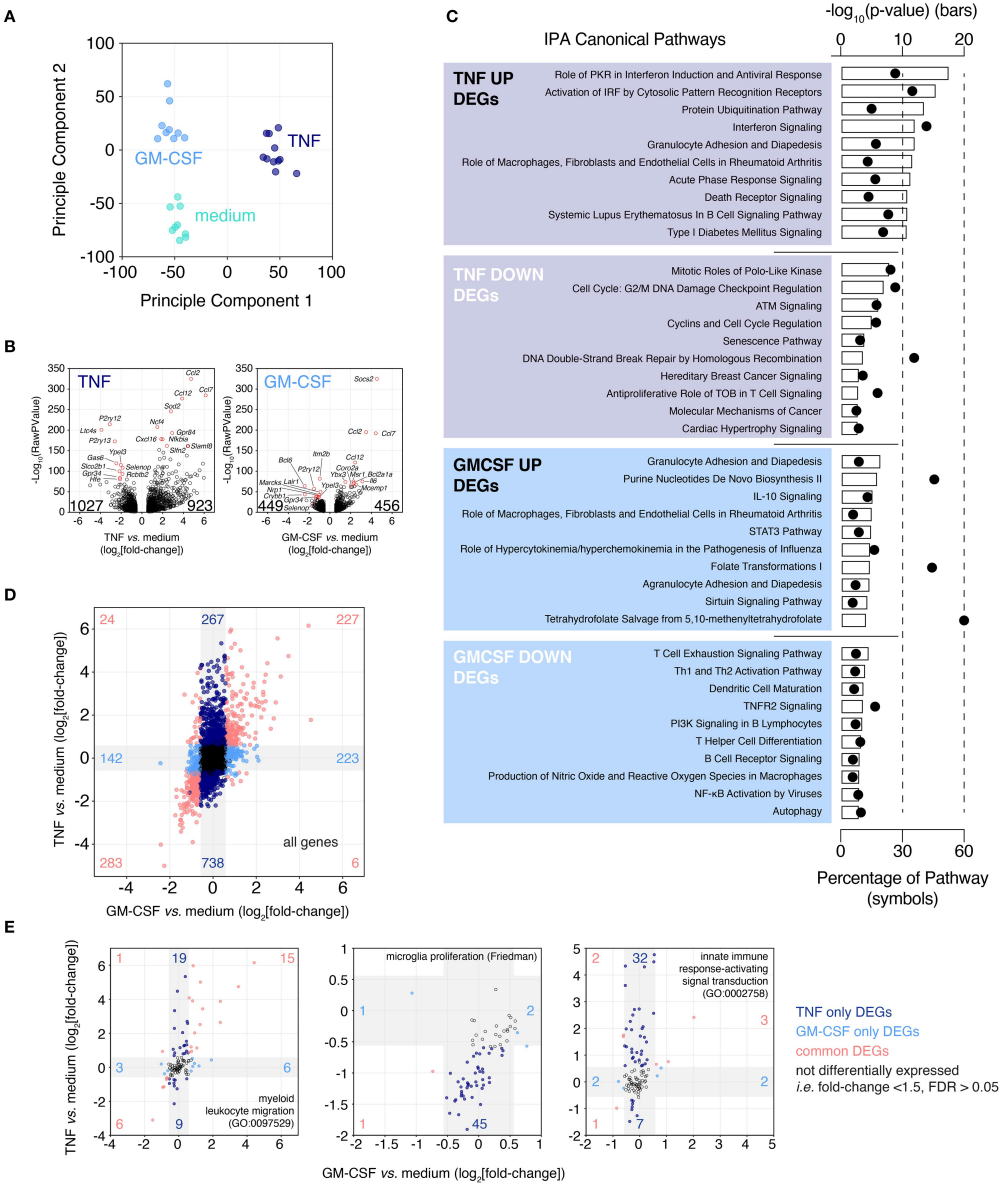
Microglia up-regulate chemotaxis/migration gene expression following exposure to cytokines associated with multiple sclerosis. Brain slices were cultured for 2 weeks then treated for 24 h with either TNF or GM-CSF. Following treatment slice microglia were isolated by FACS and expression profiling was performed by RNAseq. **(A)** Principle Component Analysis. **(B)** Volcano plots depicting TNF- or GM-CSF-induced gene expression changes. Top 10 up- and down-regulated genes ranked by FDR labeled. Total number of up- and down-regulated genes are indicated on each plot. **(C)** Ingenuity Pathway Analysis for TNF and GM-CSF treatment-induced DEGs (FDR < 0.05, absolute fold-change > 1.5). Top 10 predicted “Canonical Pathways” ranked by *p*-value shown. **(D)** Fold-change by fold-change plot comparing TNF vs. GM-CSF responses. Labeling denotes number of unique and shared, up- and down-regulated DEGs. Gray shading indicates regions of absolute fold-change < 1.5. **(E)** Fold-change by fold-change plots of TNF- vs. GM-CSF-induced changes for the indicated genesets. Gray shading indicates regions of absolute fold-change < 1.5. Labeling denotes numbers of differentially expressed genes within each region. Each microglia sample consists of two to three independently treated slices that were pooled for FACS-isolation of microglia and subsequent expression analysis by RNAseq, *n* = 9–11 samples. Pooled data from four independent experiments. See also Supplementary Figure 7 and Supplementary Tables 3, 7, 8.

To leverage this experimental system to enhance our understanding of microglia biology under neuroinflammatory conditions we assessed the effect of neuroinflammation-relevant cytokines on microglia phenotype. TNF and GM-CSF were chosen for this proof-of-concept experiment. GM-CSF/CSF2 is only expressed under pathological conditions while TNF is transcribed constitutively in microglia in human brain with the potential for further up-regulation under pathological circumstances (Olmos and Lladó, 2014; Olah et al., 2018). Different from GM-CSF, microglial-derived TNF exhibits neuroprotective properties in organotypic brain slice culture (Masuch et al., 2016a). After 2 weeks in culture brain slices were treated for 24 h with TNF or GM-CSF and microglia were isolated from cytokine-treated brain slices by FACS-sorting and gene expression profiling was performed at the population level to obtain deep coverage of gene expression changes induced by these stimuli (Figure 6).

Based on PCA and differential gene expression analysis (FDR < 0.05, absolute fold-change > 1.5), TNF was found to be the biggest driver of microglia transcriptional changes (1,950 DEGs vs. medium), compared to GM-CSF (905 DEGs vs. medium) (Figures 6A,B). Of the 923 genes up-regulated by TNF the top three most statistically significant genes were the chemokine genes *Ccl2* (up 26.8-fold), *Ccl12* (up 14.7-fold), and *Ccl7* (up 71.1-fold) (Figure 6B). One thousand twenty-seven genes were significantly down-regulated, the most significant of these were *P2ry12* (down 8.6-fold), *Ltc4s* (down 15.0-fold) and *P2ry13* (down 6.1-fold). GM-CSF-induced up-regulation of 456 genes of which the most statistically significant were *Socs3* (up 23.1-fold), *Ccl2* (up 11.3-fold), and *Ccl7* (up 21.4-fold) (Figure 6B). The GM-CSF down-regulated genes numbered 449 with the most significantly changed genes: *Itm2b* (down 2.0-fold), *Bcl6* (down 5.4-fold), and *P2ry12* (down 2.8-fold).

Due to the strong down-regulation of the microglia homeostatic gene *P2ry12* with either TNF or GM-CSF treatment we looked across a panel of consensus microglia homeostatic genes (Friedman et al., 2018) to see if these genes were similarly repressed. Across these genes we observed widespread reduction in expression especially following TNF treatment but also to a lesser extent with GM-CSF, suggesting a loss of homeostatic phenotype (Supplementary Figure 7).

To understand the likely functional consequences of these phenotypic changes induced by treatment with TNF and GM-CSF, DEGs were subjected to Ingenuity Pathway Analysis (IPA) (Figure 6C). The most highly scoring pathways for TNF up-regulated DEGs were almost all associated with inflammation (-log10 *p*-value > 10) and especially enriched for interferon signaling (#1, #2, #4 ranked hits by *p*-value). The TNF down-regulated genes were associated with proliferation or DNA damage response pathways (six of top 10), however this was less statistically significant relative to the signatures associated with the up-regulated genes (–log10 *p*-value: 8–3). Up-regulated GM-CSF responsive genes were members of pathways associated with the recruitment of blood cells to sites of inflammation (3 of top 10); other enriched pathways included IL-10 and STAT3 signaling and metabolic pathways (–log10 *p*-value: 7–4). GM-CSF down-regulated genes associated with T cell, B cell and dendritic cell activation (6 of top 10) (–log10 *p*-value: 5–3).

The relative magnitude and overlap of the TNF and GM-CSF induced gene expression changes were then considered. TNF treatment induced changes in gene expression across a large magnitude range (log_2_fold-change: −5 to +6), while the effects of GM-CSF were lesser in magnitude (log_2_fold-change: −3 to +5) (Figure 6D). The number of genes up-regulated by both TNF and GM-CSF (227 genes) or by TNF alone (267 genes) or GM-CSF alone (223 genes) were similar (Figure 6D). In contrast the number of genes uniquely down-regulated by TNF (738 genes) were far more numerous that those uniquely down-regulated by GM-CSF (142 genes). To build on our pathway analysis we compared the changes induced by TNF and GM-CSF across a curated panel of GO genesets (Supplementary Table 3). Based on the percentage of genes within each geneset that were modulated by treatment we identified “chemotaxis/migration” GO genesets as modulated by both TNF and GM-CSF (shared DEGs 10–12% vs. TNF unique DEGs 11–13%) (Figure 6E, Supplementary Table 3). In contrast the “immune activation” genesets were preferentially modulated by TNF (TNF unique 15–29% vs. shared 4–13%) as were “proliferation” genesets (TNF unique 13–17% vs. shared 1–6%) (Figure 6E, Supplementary Table 3). Neither TNF nor GM-CSF had a large effect on phagocytosis-related genes (TNF unique 9%, GM-CSF unique <1%, shared 4%).

In summary TNF provoked large changes in the microglia transcriptome that were associated with loss of homeostatic gene expression, immune activation and promotion of migration/chemotaxis as well as repression of cellular proliferation. GM-CSF promoted a loss of microglia homeostatic gene expression and also promoted migration/chemotaxis-associated gene signatures, however the changes on a per gene basis were smaller in magnitude and the smaller number of genes involved overall were almost entirely represented within the TNF response.

## Discussion

*In vitro* systems that offer *bone fide* biological insights into mechanisms associated with complex disease can inform the design and interpretation of *in vivo* studies. Here we demonstrate organotypic brain slice cultures provide a means of studying microglia in a tissue environment closely mimicking the *in vivo* brain by preserving tissue architecture and cellular composition. While the data we present are confirmatory in nature and support the use of slice cultures to allow for the maintenance of a microglia signature and function more akin to what is seen *in vivo*, we hope that these results will also serve as foundation for future studies focused on microglia biology as a valid experimental system and alternative to *in vivo* studies to generate novel biological insights. This development complements the increasing adoption of multi-cell type *in vitro* platforms such as organoids, “organ on a chip” technologies and other 3D culture techniques (Langhans, 2018; Pacitti et al., 2019; Yu et al., 2019).

In the present study, we investigated the similarity of microglia from cultured neonatal brain slices and commonly utilized *in vitro* derived microglia from dissociated and cultured neonatal brain tissue to freshly isolated adult *ex vivo* microglia. With gene expression analysis, we found distinctions between the transcriptional signatures of *in vitro*-derived microglia and *in vivo* microglia. *In vitro* microglia exhibited elevated expression of many genes that are rarely or not expressed in adult microglia, as exemplified by *Flt1* which is typically expressed in monocytes and some macrophage subsets, but not in microglia (Heng et al., 2008). In contrast when evaluating microglia identity genes, we observed low expression of these genes in the *in vitro* microglia and high expression in adult microglia. In a PCA representation these differences were most apparent in the separation between *in vitro* microglia and adult microglia along the PC1 axis. Tellingly, the most correlated genes with PC1 included well-known microglia genes such as *Hexb, Selplg, P2ry12, Cst3*, and *Tmem119*; these were highly expressed in *in vivo* microglia and rarely expressed in *in vitro* microglia. Furthermore, the *in vitro* cells exhibited high expression of gene signatures associated with macrophages, LPS and IFN-associated inflammation, as well as, neonatal microglia gene expression. Gosselin and colleagues reported the rapid loss of microglia specific gene expression of human microglia when isolated and transferred to an *in vitro* setting (Gosselin et al., 2017). Thus, the phenotype of *in vitro* cultured mouse microglia may reflect a phenomenon whereby microglia-specific gene expression is lost and replaced by undifferentiated macrophage cell gene expression. In contrast across all the data in the study, we found slice microglia consistently shared greater similarity to adult microglia (relative to *in vitro* microglia). They had higher correlated gene expression, both across the whole transcriptome and specifically for microglia identity genes, and based on pathway analysis they were less inflammatory by the 3-week timepoint, relative to 1-day. Neonatal microglia gene expression was down-regulated over time in cultured slice microglia contrasting to ongoing expression of these genes by *in vitro* microglia cells. It is established that during embryogenesis, yolk sac-derived microglia progenitors migrate early in the developing brain and colonize this tissue while neurons and astrocytes develop in their midst (Ginhoux et al., 2010). Thus, the phenotype of microglia is considered to be linked to their environment. Our data lends further support to the emerging consensus in the field that the brain slice environment conserves direct and indirect microglia interactions with other CNS cell types thereby preserving signals that maintain microglia identity and more closely modeling the *in vivo* state.

Given the organotypic brain slice culture system involves physical damage to brain tissue, we sought to determine the impact of the initial slicing event on microglia over time. This analysis allowed us to identify an optimal timepoint beyond which initial inflammatory reaction was largely resolved to mitigate any confounding influence on interpretation of interventional/treatment studies. Our results suggest release of inflammatory cytokines and loss of neurons at early timepoints post-slicing (1–3 days). Also at 1 day we observed expression of MGnD signature genes, a phenotype first characterized as the transcriptional response to apoptotic neurons (Krasemann et al., 2017). These changes were followed during the first week of culture by the up-regulation of cellular markers of inflammation on microglia such as CD68 and CD44 and an increase in the proportion of auto-fluorescent microglia likely reflective of increased clearance activities taking place in the culture system during that period of time (Burns et al., 2020). After 2 weeks of culture these markers stabilized, inflammatory soluble factors returned to baseline and the phenotype of the slice microglia most closely resembled the *in vivo* counterparts. Likewise, our Louvain clustering of the pooled slice microglia samples identified increasing numbers of microglia with a homeostatic transcriptional signature over time in culture and a relative decrease in the number of cells associated with proliferative, inflammatory and reactive clusters. We thus propose that microglia are likely activated by the apoptotic cells and cellular debris that result from slice preparation. Accordingly, this first week in culture coincides with up-regulation of the lysosomal marker CD68. These findings concur with published data reporting elevated microglia phagocytic and inflammatory activity at 4 days in culture relative to 10 days in culture (Czapiga and Colton, 1999). After 2 to 3 weeks in culture, both CD68 levels and the MGnD transcriptional signature largely subsided likely indicative of diminished microglia clearance activities in the slice and return to steady-state, which is further corroborated by the increased expression of microglia homeostatic genes observed at 3 weeks as opposed to 1 week and 1 day post-slicing timepoints. Starting at 3 weeks post-slicing however, we saw evidence of increased expression of markers associated with neurodegeneration. To avoid both the early inflammatory period and the later neurodegenerative phenotype we therefore hypothesized that 2 weeks post-slicing might correspond to the most suitable timepoint for treatment and other interventional studies aimed at understanding microglia function. Supporting this prediction, treatment of brain slices with LPS at this timepoint showed prominent similarities with the analogous *in vivo* response, establishing the brain slice culture system as a reasonable experimental approach to model complex *in vivo* microglia responses.

Similarities in cellular composition and architecture between the *in vitro* organotypic brain slice cultures and the *in vivo* setting offer advantages to model complex brain physiology and pathophysiology even though a detailed characterization of the cellular and molecular features of the brain slice culture system was lacking as it pertains to microglia properties in this experimental system. These presumed similarities prompted many studies using variants of the brain slice culture platform to study pathogenic mechanisms involved in stroke, epilepsy, neurodegenerative proteinopathies, and responses to specific neuroinflammatory stimuli (Bernardino, 2005; Vinet et al., 2012; Ziemka-Nałecz et al., 2013; Olajide et al., 2014; Hellwig et al., 2015; Gerlach et al., 2016; Masuch et al., 2016a,b; Bhatia et al., 2017; Saliba et al., 2017; Yousif et al., 2018; Araki et al., 2019; Croft et al., 2019a; Grabiec et al., 2019; Richter et al., 2019; Sheppard et al., 2019). Revisiting these studies in light of our results may reveal new insights into the role of microglia in these settings. Croft and colleagues employed AAV-mediated gene transduction approach to reproduce *bone fide* Tau neurofibrillary tangles in the slice context and then assess the impact of extended inhibition of GSK-3β on this pathology (Croft et al., 2019a). As this was observed at timepoints of 2 weeks and beyond, this would coincide with our observed expression of neurodegeneration-associated genes and provide an opportunity to study the role of microglia in this process. Kainic acid, AMPA and NMDA agonist induced excitotoxicity in brain slices have been used to model epilepsy with some studies using timepoints at 7–9 days in culture (Vinet et al., 2012; Masuch et al., 2016a,b; Araki et al., 2019; Grabiec et al., 2019) while others used later timepoints at 2–3 weeks (Bernardino, 2005; Grabiec et al., 2019). As neonatal microglia have been shown to mediated synaptic pruning with relevance to epilepsy (Andoh et al., 2019), we would wonder whether the neonatal/inflammatory phenotype of microglia observed prior to 2 weeks would alter their contribution to excitotoxicity and differ from the contribution of microglia at later timepoints where they exhibit more mature, and eventually neurodegenerative, features. These examples serve to illustrate how the data presented here provides an opportunity for a more nuanced consideration of the contribution of microglia phenotype—be it inflammatory, neonatal or neurodegenerative—on tissue pathology in slice culture models of disease.

To further our understanding of microglia phenotypic changes under inflammatory conditions, we present here preliminary data describing the microglia transcriptome after exposure to either TNF or GM-CSF, cytokines found in the multiple sclerosis demyelinating milieu (Hofman, 1989; Selmaj et al., 1991; Cannella and Raine, 1995; Vogel et al., 2015; Imitola et al., 2018). Our data at the 24 h timepoint suggested transcriptional changes that would promote immune activation, cell movement/chemotaxis and suppress proliferation. Loss of microglia homeostatic gene expression was also observed. Taken together this suggests that following exposure to TNF microglia cease homeostatic activities (including basal homeostatic proliferation), activate inflammatory signaling and contribute to the recruitment of other inflammatory cells to the site of TNF release. Although these data require substantiation and elaboration, the slice culture platform clearly shows promise as a means to study how microglia could contribute to the recruitment of CNS-resident and infiltrating cells to a developing demyelinating lesion. For example our report of TNF-induced production of CCR2 ligands *Ccl2, Ccl7* and *Ccl12* could inform follow-up studies building on reports of peripheral immune cell infiltration into the CNS during neuroinflammation (Yamasaki et al., 2014; Lagumersindez-Denis et al., 2017).

Despite progress in the characterization and uptake of the slice culture platform, limitations remain to be overcome. One such notable concern are the levels of glutamate in the culture media with potentially deleterious effects on neuronal viability. In light of this concern the current standard culture media contains horse serum, known to be lower in glutamate than serum from other species. Based on the reported levels of glutamate in horse serum (Ye and Sontheimer, 1998) we estimate the final levels of glutamate in the brain slice culture media to be ~50 μM, elevated relative to the levels observed *in vivo* (0.2–2 μM) (Hawkins, 2009). Despite this we observed viable neurons throughout the culture period, and hence speculate that astrocytes may remove glutamate from the surrounding environment consistent with published data (Ye and Sontheimer, 1998). Moreover, additional studies using medium formulations tailored to neuronal culture (BrainPhys Neuronal Medium, StemCell Technologies) did not result in any improvement in neuronal survival relative to the standard medium (data not shown). However, further study is warranted to optimize the media composition for the long-term culture of both neurons and glia.

We hope our study will provide confidence to others that this approach will provide a biologically meaningful means to further study microglia function within the native CNS microenvironment, along with methods to enable the study of large numbers of microglia at single cell resolution (e.g., flow cytometry, scRNAseq). We anticipate further improvements in the system in the future and hope that our data will provide a useful reference against which future developments can be measured. In particular additional optimization of the platform is required to maintain the “near *in vivo*” microglia phenotype beyond the 2-week culture timepoint. As this current study has focused on tissue-level and microglia changes, additional contributions of experts in neuronal, astroglial, oligodendroglial and endothelial cell biology will be required to further characterize the phenotype of these cells within the organotypic slice culture system. These contributions will be essential for this technology to reach its full potential as a means to uncover novel insights into steady state and disease-associated CNS biology.

## Data Availability Statement

The datasets presented in this study can be found at https://www.ncbi.nlm.nih.gov/geo/query/acc.cgi?acc=GSE154855.

## Ethics Statement

The animal study was reviewed and approved by Biogen Institutional Animal Care and Use Committee (IACUC).

## Author Contributions

AD, CB, and MM: conceptualization. AD, MB, JB, CB, and MM: methodology. AD and MF: software. AD, RC, NR, PC, TC, ML, and KE: investigation. CR, CS, and NA: project administration. AD, CB, MM, DH, NF, RR, and H-HT: supervision. AD, CB, and MM: writing – original draft. AD, CB, MM, RR, JB, DH, and NF: writing – review & editing. All authors contributed to the article and approved the submitted version.

## Conflict of Interest

AD is employed by Putnam Associates; MB, DH, JB, RC, PC, TC, KE, CS, H-HT, NA, NF are full-time employees and shareholders of Biogen; RR is employed by Third Rock Ventures; CR is employed by Genomics Institute of the Novartis Research Foundation; and MF is employed by bluebird bio. The authors declare that this study was funded by Biogen and designed and executed by current or former Biogen employees.
