# Supplementary material for: Organotypic Brain Slice Culture Microglia Exhibit Molecular Similarity to Acutely-Isolated Adult Microglia and Provide a Platform to Study Neuroinflammation
Source: Front Cell Neurosci. 2020 Dec 21;14:592005. doi: 10.3389/fncel.2020.592005 (PMC7812919; doi:10.3389/fncel.2020.592005)

Delbridge et al. – Supplementary Information

Supplementary Figures (included at the end of this document)

Supplementary Figure 1: Preparation of neonatal brain slices for culture. Schematic depiction of the steps performed to collect brain slices for *in vitro* culture. (1) Brain isolated from neonatal mice. (2) Removal of olfactory bulb and cerebellum. (3) Mounting of tissue and preparation of 300 µm slices. (4) Collection of slices for culture. See methods for detailed description.

Supplementary Figure 2: Changes in cell marker gene expression over time in culture. Overall expression changes (z-score normalized) for validated astrocyte, oligodendrocyte precursor cell (OPC), oligodendrocyte, and endothelial cell marker genes (McKenzie et al., 2018) in cultured brain slices were plotted over time. Geneset mean +/- SD, n=3 slices per timepoint. See also Figure 1.

Supplementary Figure 3: Identification of slice microglia by flow cytometry and protein marker expression over time in culture. (Upper panel) Adult *ex vivo* microglia, and slice microglia freshly isolated ("fresh slice") or after 1 week or 3 weeks in culture were identified for flow cytometric analysis based on expression of CD11B and CD45. (Lower panel) Percentage of auto-fluorescent microglia over time in culture. Each symbol represents microglia data from an individual slice (n=14-28), bars indicate mean +/- SD. Representative FACS plots shown. See also Figure 2.

Supplementary Figure 4: Microglia isolated from cultured brain slices exhibit reduced protein expression of microglia markers. CX3CR1, P2RY12 and TMEM119 were characterized at the protein level on slice microglia over time in culture by flow cytometry. Mean +/- SD shown, data pooled from 4 independent experiments, n=3-10 independent slice microglia samples. **** and * denote adjusted p-value less than 0.0001 and 0.05 by one-way ANOVA, respectively. See also Figure 3.

Supplementary Figure 5: Genes down-regulated in microglia isolated from cultured brain slices and *in vitro* primary microglia relative to adult microglia show limited pathway enrichment. Brain slice culture, *in vitro* primary/mixed glial culture and freshly-isolated adult microglia were isolated by FACS and subjected to single cell RNA expression profiling. Down-regulated differentially expressed genes (DEGs) between either slice 3 weeks or *in vitro* microglia and adult microglia were compared and subjected to Ingenuity Pathway Analysis to identify enrichment of the DEGs in canonical pathways. Top pathways ranked by p-value shown for DEGs shared or unique to slice 3 weeks and *in vitro* microglia, relative to adult microglia. See also Figure 3.

Supplementary Figure 6: Clustering of brain slice, *in vitro* and adult microglia. Brain slice culture, *in vitro* primary/mixed glial culture and freshly-isolated adult microglia were isolated by FACS and subjected to single cell RNA expression profiling. (Upper left) UMAP plot of Louvain clustering. (Upper right) UMAP plot annotated with slice microglia sample identity. (Middle left) UMAP plot annotated with adult microglia. (Middle right) UMAP plot annotated with *in vitro* microglia. (Lower panel) UMAP plot with slice microglia cluster annotation. See also Figure 4.

Supplementary Figure 7: Microglia lose microglia identity gene expression following exposure to cytokines associated with neuroinflammation. Brain slices were cultured for 2 weeks then treated for 24 hours with either TNF and GM-CSF. Following treatment slice microglia were isolated by FACS and expression profiling was performed by RNAseq. Relative expression of microglia homeostatic genes (Friedman et al. 2018). Each column represents data from an independent microglia sample. Scale represents median absolute deviation by row (see Methods). Each microglia sample consists of 2-3 independent slices that were treated then pooled for FACS-sorting of microglia and expression analysis by RNAseq, n=9-11 samples. Pooled data from 4 independent experiments. See also Figure 6.

Supplementary Tables (included as separate files)

Supplementary Table 1: Brain slice microglia gene expression enrichment analysis by Louvain cluster and Ingenuity Canonical Pathway Analysis. Genes enriched by cluster relative to the pooled cells of all other clusters were determined and then subjected to IPA Canonical Pathway Analysis.

Supplementary Table 2: LPS induced differentially expressed genes and GO term analysis unique to either brain slice microglia or acutely isolated adult microglia. LPS microglia DEGs induced in either brain slices or *in vivo* were determined and compared to identify up- and down-regulated DEGs that were unique to either context. These up- and down-regulated, slide unique and *in vivo* unique DEGs were further characterized by GO term analysis.

Supplementary Table 3: TNF and GM-CSF differentially expressed gene enrichment within a focused set of Gene Ontology genesets. The number of TNF and GM-CSF differentially expressed genes (DEGs)

within a focused set of immune activation, migration/chemotaxis, proliferation and phagocytosis GO pathway genesets was determined including the number of unique and shared DEGs between the two stimuli.

Supplementary Table 4: Pearson correlation analysis of scRNAseq PCA data to determine genes associated with PC1 and PC2. Brain slice culture, *in vitro* primary/mixed glial culture and freshly-isolated adult microglia were FACS-sorted and subjected to single cell RNA expression profiling and Principle Component Analysis (see Figure 3). Pearson correlation analysis of genes correlated with PC1 and PC2.

Supplementary Table 5: Ingenuity Canonical Pathway Analysis of differentially expressed genes shared (common) to both or unique to either 3 week brain slice microglia and *in vitro* derived microglia, relative to adult *ex vivo* microglia.

Supplementary Table 6: Ingenuity Canonical Pathway Analysis of *in vitro*-derived microglia differentially expressed genes relative to 3 week brain slice microglia.

Supplementary Table 7: Differentially expressed genes in isolated brain slice microglia following exposure of cultured brain slices to either TNF or GM-CSF for 24 hours.

Supplementary Table 8: Ingenuity Canonical Pathway Analysis of differentially expressed genes in isolated brain slice microglia following exposure of cultured brain slices to either TNF or GM-CSF for 24 hours.

Supplementary Figure 1

Delbridge et al.

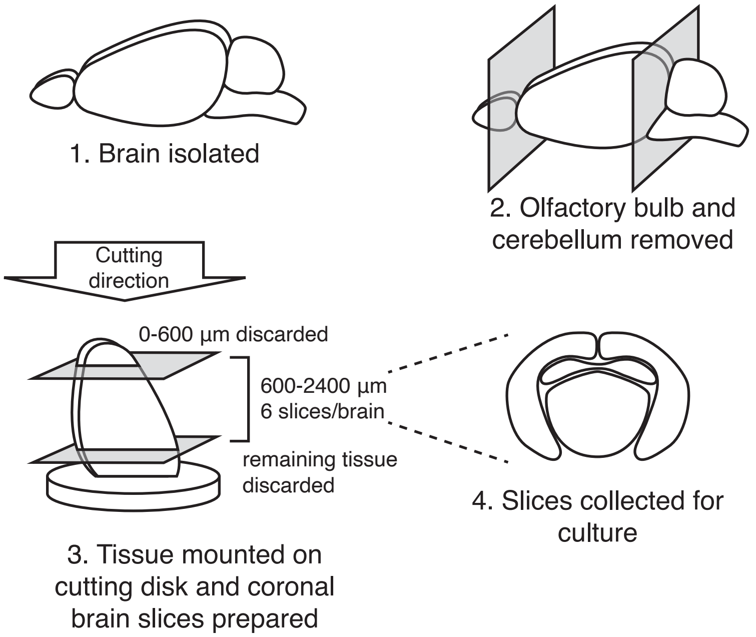

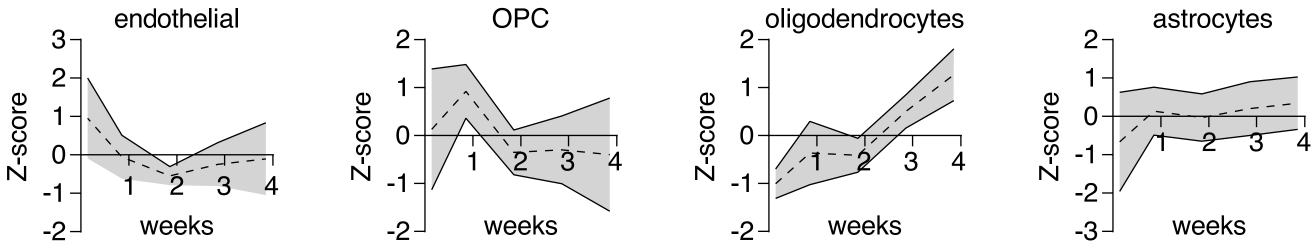
endothelial
OPC
oligodendrocytes
astrocytes
Z-score
weeks
3
2
1
0
-1
-2
-3
1
2
3
4

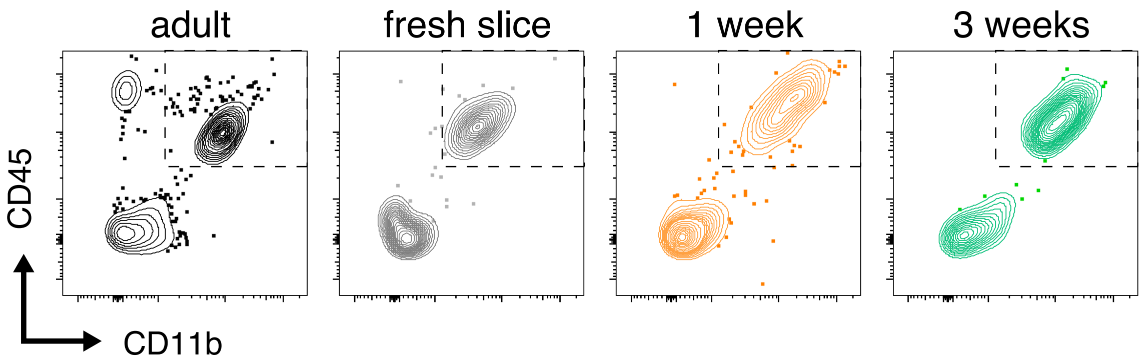
adult
fresh slice
1 week
3 weeks
CD45
CD11b

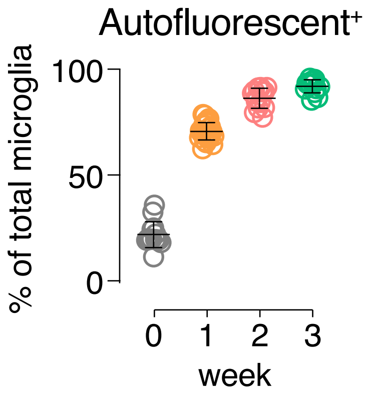
Autofluorescent+
% of total microglia
100
50
0
0
1
2
3
week

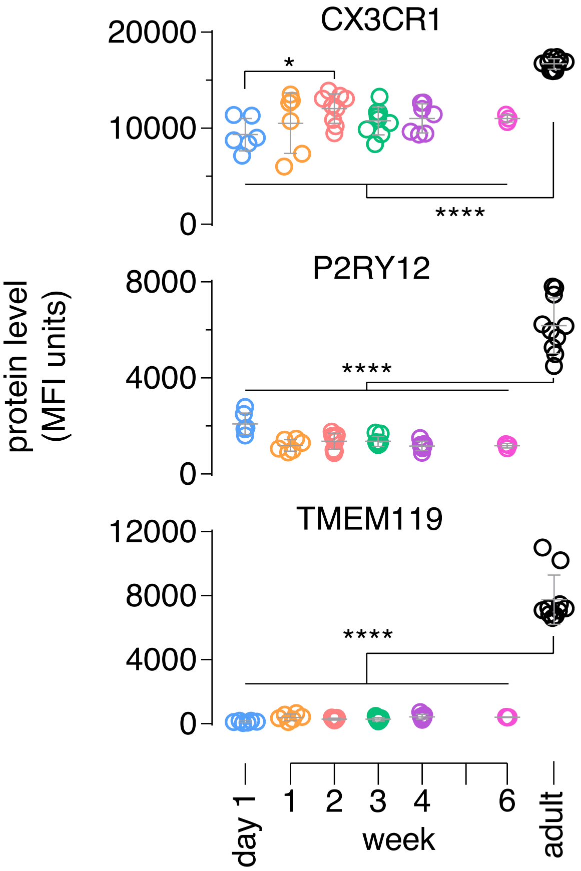
CX3CR1
20000
10000
0
*
****
P2RY12
8000
4000
0
****
protein level
(MFI units)
TMEM119
12000
8000
4000
0
****
day 1
1
2
3
4
6
week
adult

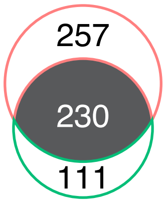

in vitro unique canonical pathways	$-log_{10}$(p-value)
Gap Junction Signaling	3.8
PI3K Signaling in B Lymphocytes	3.3
Oxidative Phosphorylation	3.1
GPCR-Mediated Integration of Enteroendocrine Signaling Exemplified by an L Cell	3.1
Glioblastoma Multiforme Signaling	2.9
Role of NFAT in Regulation of the Immune Response	2.6

shared canonical pathways	$-log_{10}$(p-value)	
	in vitro	slice
T Helper Cell Differentiation	6.4	5.3
Altered T Cell and B Cell Signaling in Rheumatoid Arthritis	5.7	4.7
T Cell Exhaustion Signaling Pathway	5.3	3.6
B Cell Development	4.7	3.5
Th1 and Th2 Activation Pathway	4.5	3.7
Graft-versus-Host Disease Signaling	4.1	3.0
Hepatic Fibrosis / Hepatic Stellate Cell Activation	2.8	3.5

slice unique canonical pathways	$-log_{10}$(p-value)
Inflammasome pathway	5.7
MSP-RON Signaling Pathway	4.4
dTMP De Novo Biosynthesis	4.0
Urea Cycle	3.3
TWEAK Signaling	3.2
TR/RXR Activation	3.2

Supplementary Figure 6 Delbridge et al.

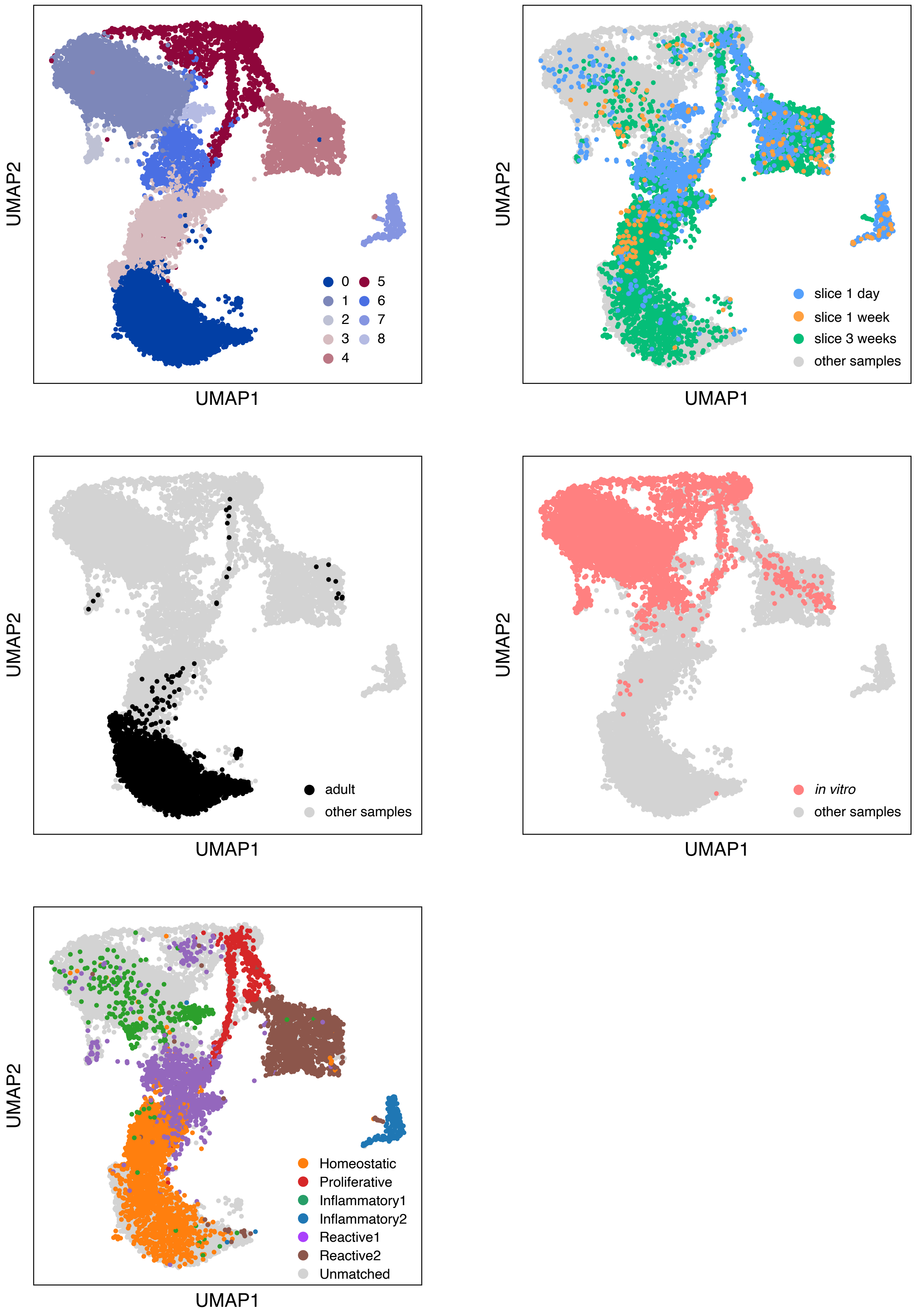

Supplementary Figure 7

Delbridge et al.

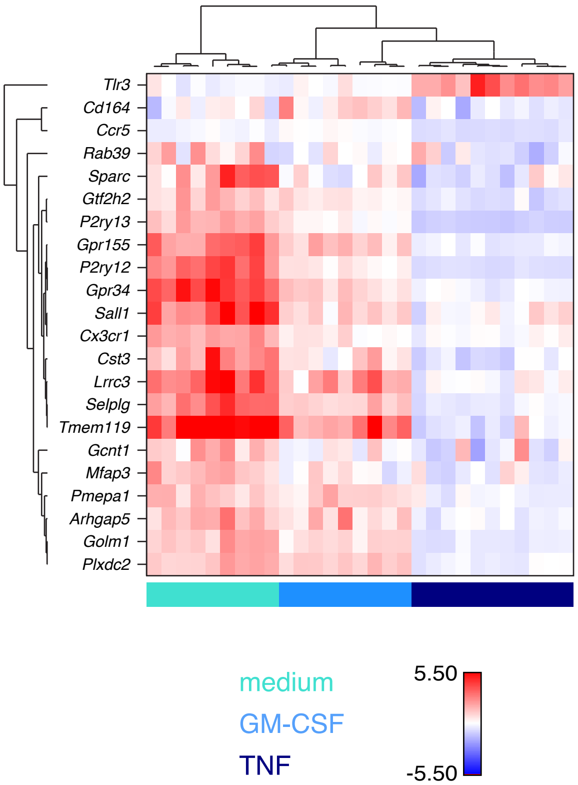

Supplement: Supplementary file 9 [file Image_1.pdf]
